# Advancements in Aptamer‐Driven DNA Nanostructures for Precision Drug Delivery

**DOI:** 10.1002/advs.202401617

**Published:** 2024-05-07

**Authors:** Moein Safarkhani, Sepideh Ahmadi, Hossein Ipakchi, Mohammad Reza Saeb, Pooyan Makvandi, Majid Ebrahimi Warkiani, Navid Rabiee, YunSuk Huh

**Affiliations:** ^1^ NanoBio High‐Tech Materials Research Center Department of Biological Sciences and Bioengineering Inha University 100 Inha‐ro Incheon 22212 Republic of Korea; ^2^ School of Chemistry Damghan University Damghan 36716‐45667 Iran; ^3^ Department of Chemical Engineering McMaster University Hamilton L8S 4L8 Canada; ^4^ Department of Pharmaceutical Chemistry Medical University of Gdańsk J. Hallera 107 Gdańsk 80‐416 Poland; ^5^ The Quzhou Affiliated Hospital of Wenzhou Medical University Quzhou People’s Hospital 324000 Quzhou Zhejiang China; ^6^ Centre of Research Impact and Outreach Chitkara University Rajpura Punjab 140417 India; ^7^ Department of Biomaterials Saveetha Dental College and Hospitals SIMATS Saveetha University Chennai 600077 India; ^8^ School of Biomedical Engineering University of Technology Sydney Ultimo NSW 2007 Australia; ^9^ Institute for Biomedical Materials and Devices (IBMD) University of Technology Sydney Sydney NSW 2007 Australia; ^10^ Centre for Molecular Medicine and Innovative Therapeutics Murdoch University Perth WA 6150 Australia

**Keywords:** aptamers, DNA nanostructures, DNA origami, DNA Tetrahedral, key‐like ligand of nanocarrier

## Abstract

DNA nanostructures exhibit versatile geometries and possess sophisticated capabilities not found in other nanomaterials. They serve as customizable nanoplatforms for orchestrating the spatial arrangement of molecular components, such as biomolecules, antibodies, or synthetic nanomaterials. This is achieved by incorporating oligonucleotides into the design of the nanostructure. In the realm of drug delivery to cancer cells, there is a growing interest in active targeting assays to enhance efficacy and selectivity. The active targeting approach involves a “key‐lock” mechanism where the carrier, through its ligand, recognizes specific receptors on tumor cells, facilitating the release of drugs. Various DNA nanostructures, including DNA origami, Tetrahedral, nanoflower, cruciform, nanostar, nanocentipede, and nanococklebur, can traverse the lipid layer of the cell membrane, allowing cargo delivery to the nucleus. Aptamers, easily formed in vitro, are recognized for their targeted delivery capabilities due to their high selectivity for specific targets and low immunogenicity. This review provides a comprehensive overview of recent advancements in the formation and modification of aptamer‐modified DNA nanostructures within drug delivery systems.

## Graphical abstract

The review explores developments of aptamer‐driven DNA nanostructures for targeted drug delivery. DNA nanostructures, featuring tailorable platforms, integrate oligonucleotides to orchestrate molecular arrangements. Active targeting assays improve therapeutics delivery to cancerous cells by a “key‐lock” mechanism. DNA nanostructures efficient cell membrane penetration, and provide cargo delivering to the nucleus. Aptamers, with low immunogenicity and high selectivity, play an undeniable role in site‐specific delivery.

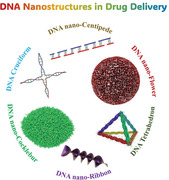

## 1 DNA Nanostructures

DNA nanostructures, constituted by DNA, serve as both structural and functional elements. The prevalent B‐form structure of DNA manifests as a double‐stranded helix measuring ≈2 nm (diameter), with 3.4 nm (or 10.4 base pairs) per helical turn. B‐DNA exhibits a persistence length of ≈50 nm or 150 base pairs, thereby imparting to DNA a rather stiff polymer characteristic at the nanoscale.^[^ ^1^ ^]^ Through Watson–Crick base pairing interactions, DNA can autonomously self‐assemble into the B‐DNA configuration, relying on complementary adenine (A) and thymine (T), as well as cytosine (C) and guanine (G) pairs. Furthermore, these DNA nanostructures can serve as scaffolds, thereby facilitating the construction of more intricate and sophisticated molecular architectures.^[^ ^2^ ^]^ Structural DNA nanotechnology distinguishes itself from other molecules by its predictable and programmable interactions. DNA manifests noteworthy binding specificity and thermodynamic stability, affording malleability for modifications via chemical assays or enzymatic processes that induce alterations in its structure.^[^ ^3^ ^]^ Despite the inherent intricacies characterizing the DNA structure, a steadfast adherence to the physiochemical principle aimed at optimizing base‐pairing events perseveres. This dedication, in consequence, leads to a concomitant reduction in the thermodynamic free energy across the entire self‐assembly system.^[^ ^4^ ^]^ The employment of modular, branched building blocks not only facilitates the creation of elaborate, higher‐order structures but also contributes to the nuanced development of such formations. The noncovalent and reversible attributes inherent in DNA hybridization present an avenue for the discerning selection of conditions, thereby guiding the self‐assembly trajectory with precision. Consequently, this strategic control fosters both the expeditious and proficient fabrication of nanostructures according to predefined specifications.^[^ ^5^ ^]^ In addition, the seamless integration of dynamic features, like reversible assembly, unidirectional motion, and reconfiguration can be accomplished through a strand displacement mechanism that operates without the involvement of enzymes. Taken as a whole, Structural DNA nanotechnology furnishes an extensive design framework within the realm of nanofabrication, establishing itself as the fundamental cornerstone for a myriad of applications. This pioneering methodology not only facilitates the creation of intricately organized 2D and 3D DNA nanostructures across scales ranging from nanometers to millimeters but also manifests a broad spectrum of diverse shapes and configurations. Consequently, this approach represents a significant advancement in the ability to engineer sophisticated and versatile nanoscale structures.^[^ ^4^, ^6^ ^]^

DNA nanotechnology can use small strands of DNA to form different 1D‐ 3D nanostructures. These nanostructures have several advantages, such as ease of functionalization, and great capability to encapsulate different nanoscale cargo. However, the applications of these structures have been restricted to biological applications due to a lack of studies of these structures in vivo systems.^[^ ^7^ ^]^ 1D self‐assembled DNA nanostructures showed nanowire shape structure.^[^ ^8^ ^]^ 2D and 3D nanostructures have effective regions for molecular fixation. As a result, by changing the signal molecules at specified points, their movement can be controlled, so it is possible to better study intermolecular interactions. Studies showed different advantages of the 3D DNA nanostructures than ss DNA and dsDNA to degradation. In general, it seems that 3D DNA nanostructures with modified bases resist the high temperatures, enhanced stability, and nuclease established in the fluids.^[^ ^5^, ^9^ ^]^ These factors might apply as strategies for the fabricating of DNA nanostructures for biomedical uses.

Ensuring the stability of DNA nanostructures under biological conditions is a critical aspect of their design. The biochemical environment and inherent properties of DNA molecules play a crucial role in enhancing stability and resilience, particularly in aqueous and non‐frozen states. The stability of DNA structures at 37 °C, with a significant consideration for magnesium cation concentration, is vital for DNA duplex stabilization.^[^ ^10^ ^]^ The first point is canning temperature changes cause disturbance in the assembly of these structures. The DNA melting temperature is greater compared to the RT if the hybridization area is longer than 12 bases. The results indicate that preserving DNA at 4 °C demonstrates enduring stability over time, particularly when combined with an elevated DNA concentration or the inclusion of carrier DNA or RNA, thereby mitigating degradation rates. Mechanisms of degradation, including oxidation, hydrolysis, and depurination, emphasize the importance of pH regulation for stability. The hydrolysis rate of DNA is significantly influenced by both acidic and basic conditions. Both cross‐linking agents and the use of chemically modified DNA bases, including locked nucleic acids (LNAs), can enhance heat tolerance in DNA nanostructures.^[^ ^11^ ^]^ LNAs are distinguished by the inclusion of a strategically positioned methylene bond connecting the 2′‐OH and 4′‐carbon of the ribose, a structural characteristic that significantly augments thermal resilience within DNA nanostructures. The synthesis of DNA origami within conventional buffers, notably those enriched with divalent cations such as magnesium ions (Mg^2+^), assumes a pivotal role in mitigating repulsive electrostatic interactions arising from the inherent negative charge inherent to the DNA phosphate backbone.^[^ ^12^ ^]^ The failure to address these electrostatic forces poses a potential threat to the stability of intricately designed DNA nanostructures. The challenges become particularly pronounced when confronting the dilution of DNA nanostructures in fluidic environments and managing fluctuations in Mg^2+^ concentration, thereby adding a layer of intricacy to the process. Notably, the concentration of Mg^2+^ within these buffers surpasses the typical levels found in physiological buffers, spanning the range of 5 to 30 millimolar (mM). Certain structural configurations, as exemplified by wireframe DNA structures such as nanocubes, tetrahedra, and polyhedral meshes, exhibit a notable resilience against reductions in Mg^2+^ concentration. In striking contrast, DNA origami structures display heightened sensitivity to reductions in Mg^2+^ concentration (≤ 1 mM), necessitating an elevated level of magnesium ions to uphold their structural integrity. This underscores the intricate interplay of factors influencing the stability and functionality of diverse DNA nanostructures under varying conditions within the realm of nanotechnology.^[^ ^13^ ^]^ Besides, these structures are sensitive to diminution since the dense packing of the helices needs a great concentration of Mg^2+^ concentration. However, the existence of phosphate ions and EDTA can help DNA origami denaturation. EDTA can enable DNA origami denaturation via chelating and eliminating Mg^2+^ ions from the backbone; in fact, EDTA interacts with the Mg^2+^ ions and prompts DNA origami denaturation by decreasing the strength of the Mg^2+^ and DNA interaction.^[^ ^14^ ^]^ The shape of DNA origami structures significantly affects their sensitivity to Mg^2+^ diminution; long origami nanotubes maintain stability, while octahedra and rods assume an amorphous state at room temperature. However, some studies showed that by choosing reasonable buffers and taking into account superstructure effects, the DNA origami's integrity can be preserved at low micromolar Mg^2+^ concentration for a long time.^[^ ^14a^ ^]^

Additionally, shorter DNA sequences that hybridize near room temperature may lack adequate sequence specificity within extensive, various libraries of DNA strands—common components in almost all DNA storage systems. DNA stored at −20 °C can persist moderately stable for a long time, and preserving a high concentration of DNA can help slow down degradation rates.^[^ ^15^ ^]^ In addition to temperature which affects the degradation rate of DNA strands, pH control can be the next significant factor for enhancing the stability of DNA. The integrity and stability of DNA structures stay at a physiological pH of 7.0. Both basic and acidic environments can increase the degradation rate of DNA by enhancing the DNA's electrophilicity. With pH changes toward acidity (pH 4.0 to 6.0), the rate of DNA degradation increases greatly.^[^ ^14b^ ^]^ Some studies have shown that a 90‐min exposure to pH 4.0 results in almost complete degradation of the DNA sample.^[^ ^16^ ^]^ Therefore, precise control of pH, along with careful management of exposure duration to elevated temperatures, is crucial in the design considerations for storage systems.^[^ ^5a^ ^]^ Utilizing common buffers like TE and PBS can assist in maintaining pH levels at targeted thresholds. Furthermore, the inclusion of additives, such as DNA‐stable TM, salts, and trehalose, may provide protective effects in DNA storage systems.^[^ ^5a^ ^]^

An imperative consideration lies in the formidable challenge presented by enzymes responsible for the degradation of DNA nanostructures, notably within blood and serum, thereby complicating their efficacious delivery.^[^ ^17^ ^]^ The ubiquitous existence of nucleases in bodily fluids, encompassing blood, urine, and saliva, poses a substantial impediment as they catalyze the degradation of DNA through the hydrolysis of phosphodiester bonds.^[^ ^18^ ^]^ Nucleases, pivotal in biological procedures like DNA repair, replication, recombination, and structural modifications of nucleic acids, occupy a crucial role.^[^ ^17^, ^19^ ^]^ Given the diverse array of nucleases present in both blood and fetal bovine serum (FBS), researchers commonly employ laboratory settings to assess the potential impact of nucleases on the efficacy of DNA‐based structures across diverse biological properties.^[^ ^20^ ^]^ Despite the indispensability of nucleases in pivotal biological functions, their interference with the DNA nanostructures’ stability underscores the exigency of comprehensive investigations into biostability under in vivo conditions or those simulating physiological body fluids. Empirical studies elucidate that DNA origami and nanostructures maintain stability for a duration ranging from 2 to 8 h within environments containing FBS. Although the enzymatic activity in human serum is notably lower than that observed in FBS. In one study, DNA origami nanostructure (5 nM) was incubated for 0.25 to 24 h in RPMI medium containing 10% FBS, and notable degradation of DNA nanostructure was detected after 2 h.^[^ ^21^ ^]^ This fast degradation is triggered by the high nuclease activity of FBS. However, in human serum, 6 times longer lifetimes have been detected.^[^ ^22^ ^]^ Researchers assessed the wireframe DNA origami's stability by incubating the nanostructure at 37 ˚C in the PBS buffer containing different concentrations of DNase I (≈1–57 U mL^−1^). They showed the stability of DNA nanostructure for 28 U  mL^−1^ of DNase I for an hour.^[^ ^23^ ^]^ Although, at 3.6 U mL^−1^ of enzyme in the human blood, the samples exhibited low degradation for up to 12 h incubation.^[^ ^24^ ^]^ In another study, Castro and co‐workers showed that DNase I is the most plentiful nuclease in blood and serum. However, DNase‐I‐prompted degradation was slower for the DNA origami than for ds DNA.^[^ ^25^ ^]^ However, the time scales of enzymatic‐induced DNA nanostructure degradation, both in serum/blood or in culture medium, strongly depend on experimental conditions and temperature, and different mechanisms are needed to increase the lifetime of nanostructures for medical applications.

Recently, different strategies were developed for moderate nuclease resistance at the design phase, such as coating of the DNA nanostructure, modification, solution treatment, etc., which is shown in **Table** **1**. The nuclease's effect on the DNA structures was decreased via the use of inhibitors during the formation of DNA structures. For instance, in RPMI medium incubated DNA origami contained with 10% FBS degraded entirely during a day. The FBS heating up to 75 °C for 5 min before pouring into the cell culture medium protracted the DNA structure, according to the inactivation of nucleases. However, heating the FBS can affect serum proteins and cell growth.^[^ ^21^ ^]^ In other cases, some compounds can limit admittance to the minor groove of dsDNA and may prevent the activity of DNase I. Researchers described the effect of various minor‐groove binders on diminishing the DNA origami's nuclease degradation in another study. The most effective stabilizer was Diamine 2‐(4‐amidinophenyl)−1H‐indole‐6‐carboxamidine (DAPI), enhancing the structures’ protection in 10% mouse serum to one day.^[^ ^26^ ^]^

**Table 1:** Some strategies are applied for nuclease resistance in various DNA nanostructures.

Strategies	Types	DNA nanostructures	Mechanisms	References
Chemical modifications	L‐DNA	DNA Tetrahedron	2′‐O‐Me modification in siP53 can provide sufficient stability for in vivo application against DNase I, and RNase A enzymes	[30]
	Crosslinking (click chemistry)	Nanotube	Increased resistance of DNA nanostructure against degradation and high temperature	[31]
	Crosslinking (UV‐induced T–T dimers)	DNA origami	Situation of thymidine's inside DNA nanostructures and formation of cyclobutane pyrimidine dimer (CPD) bonds permits stability of DNA nanostructures in high temperature (up to 90 °C) and enzymatic degradation.	[32]
Coatings strategies	Peptoids (PE2)	Octahedral DNA origami	The decreased number of Octahedral DNA origami was detected after incubation at 37 °C for 24 h	[27]
	PEGylated lipid bilayer	DNA octahedron	DNA octahedron Envelopment in PEGylated lipid bilayers deliberated protection from DNase I nuclease digestion	[28]
	Polysaccharides (chitosan and linear polyethyleneimine)	wireframe DNA origami	Agarose gel electrophoresis analysis exhibited the degradation of DNA origamis after 24 incubations in the existence of DNase I (10 U mL^−1^)	[33]
Solution treatment	FBS heat treatment	Octahedron origami	Heat inactivation at 75 °C for 5 min inactivated and inhibited nuclease activity	[21]
	Minor‐groove binders (DAPI)	DNA origami	DAPI can enhanced the protection of nanostructures in mouse serum (10%) for a day	[26]
	DAPI	wireframe DNA origami	Long‐term stability of two‐helix wireframe structures was detected more 24 h	[34]

Coating of DNA nanostructures non‐covalently with other molecules via electrostatic interactions, atomic layer deposition (ALD), and mineralization can be used to stabilize DNA origami nanostructures from degradation. For example, Wang and co‐workers established the DNA structures in cell media via incubating at 37 °C for a day in the DMEM and RPMI medium with low Mg^2+^ concentration. In the existence of 10% FBS combined with the DMEM, the coated structures based on peptoids decreased in number after 24 h^[^ ^27^ ^]^ (Table 1). Another study showed that DNA octahedron envelopment in PEGylated lipid bilayers deliberated protection from nuclease digestion and enhanced the structures’ percentage from 30% up to 85% after incubating in 20 U DNase I for a day.^[^ ^28^ ^]^ Researchers show that glutaraldehyde cross‐linking of PEGylated oligolysine‐coated DNA nanostructures prolongs survival by up to 250‐fold to 48 h throughout incubation with 2600 times the concentration of DNase I. This assay offers a potential method for protecting nanostructures both in vivo and in vitro.^[^ ^29^ ^]^ These approaches have been established to enhance the nuclease resistance of nanostructures, although holding their activity, and the stability of different nanostructures has been studied in biological fluids.

Methodologies involving the assembly of DNA into 3D configurations and the scrutiny of structural integrity under diverse conditions, including denaturation, have been pursued. Remarkably, the tetragonal stability of the wireframe manifests a 50‐fold augmentation relative to double‐stranded DNA (dsDNA) under ambient temperature conditions. The progression of DNA carriers signifies considerable potential within drug delivery applications, particularly in realizing targeted drug release, notwithstanding the inherent challenge associated with delivering these molecules to specific lesion sites.^[^ ^18^, ^35^ ^]^ The maintenance of stability in these carriers within the environmental milieu assumes paramount importance for their optimal functionality. DNA, serving as the repository of genetic information, exhibits notable biocompatibility and design flexibility, extending its utility beyond the established role in genetic coding.^[^ ^4^, ^36^ ^]^ Within the field of drug delivery, DNA assumes a multifaceted role, serving as a carrier for pharmaceutical agents, a component for target recognition, and notably, as the therapeutic substance itself.^[^ ^37^ ^]^ In addition to the extensively documented double‐helix structures, DNA exhibits a proclivity for adopting non‐canonical configurations, including but not limited to triplexes, hairpins, cruciforms, G‐quadruplexes, and bulges.^[^ ^38^ ^]^ Noteworthy advancements have been observed in this domain with the emergence of DNA nanotechnology, culminating in the intricate design and fabrication of nanostructure res such as DNA origami, nanoflowers, cages, nanotubes, and polyhedrons.^[^ ^39^ ^]^ These structures not only facilitate effective drug loading through a diverse array of mechanisms but also induce responsive drug release via structural modifications in the DNA framework.

DNA nanostructures were synthesized by different methods, such as DNA Origami technique, Rolling Circle Amplification (RCA), and DNA Brick Assemblies. DNA Origami technology includes folding an ssDNA scaffold with hundreds of short‐staple strands to form 2D and 3D nanostructures.^[^ ^40^ ^]^ RCA technology is the potential method based on the enzymatic amplification of circular DNA sequences to produce long ssDNA scaffolds that can be applied to building DNA nanostructures.^[^ ^41^ ^]^ This technique was applied for the synthesis of different DNA nanostructures, like Nanotubes, DNA Nanowires, DNA origami structures, nanoflowers, and nanoribbon structures with highly detailed control over their shapes size, and functionality.^[^ ^41^, ^42^ ^]^ These RCA‐based DNA nanostructures can be applied as scaffolds or carriers to load with different metal nanoparticles, biomolecules, or drugs.^[^ ^43^ ^]^ In DNA Brick Assembly 3D DNA nanostructures can be formed using short synthetic DNA strands named “DNA bricks.” These bricks are considered to self‐assemble into specific configurations via base‐pairing interactions. Some of the various DNA nanostructures could be prepared using DNA Brick Assembly including DNA nanocages and DNA Nanotubes.^[^ ^44^ ^]^ By arranging DNA bricks in a detailed spatial procedure, researchers can form nanocages with distinct shapes and sizes, appropriate for encapsulating biomolecules with high capacity.^[^ ^45^ ^]^ Each of these techniques has its benefits and limitations, and the selection of methods depends on the structure and application of the DNA nanostructures. Designing various shapes of DNA nanostructures permits flexibility in uses and functionality. The shape of the DNA nanostructure can affect its stability, binding affinity, specificity, and types of applications. For example, DNA structures can be formed in different forms, such as linear or branched structures, or 3D nanostructures, with each shape proposed unique properties. Linear DNA structures may be appropriate for binding assays, although branched DNA structures can be applied for drug delivery.^[^ ^46^ ^]^ Branched DNA nanostructures have a high surface area for drug loading and can potentially deliver a greater drug than 3D DNA nanostructures. For example, Y‐shape DNA origami modified with ZnFs can be used for the release of PTEN tumor suppressor proteins to prevent tumor growth. This functional Y‐DNA nanostructure displayed self‐assembled structures with high loading capacity and strong resistance to the exonuclease activity.^[^ ^46a^ ^]^ Polypod‐like structure is a type of branched nano‐assemblies comprised of a structural body “trunk” collected with many “legs.” In comparison with linear structures, the multi‐legged DNA nanostructures’ backbone offers stability in structure, and the branched structure significantly enhances drug binding targets.^[^ ^47^ ^]^

Although, 3D DNA nanostructures have precise control over their morphology and size, which can be useful for targeted drug delivery. They can also be designed to encapsulate drugs and protect them from degradation until they reach the target site. Besides, they can be modified with ligands to enhance their efficiency in delivering drugs to targeted tissues.^[^ ^48^ ^]^ In this study, the focus is more on 3D DNA nanostructures for targeted drug delivery. 3D DNA origami includes folding a long ssDNA molecule into 3D structures using short “staple” strands to embrace the structure together. They have several advantages for targeted drug delivery, such as high loading capacity, high stability and biocompatibility, and high potential for modification with different ligands, such as aptamers to achieve a targeted drug delivery.^[^ ^49^ ^]^ In one study, Liu and co‐workers proposed an assay to increase the transmembrane ability of DNA origami sheets by changing their configuration from 2D to 3D structures in a tumor model. These results offer promising visions for DNA nanostructures’ further designs for transmembrane delivery.^[^ ^50^ ^]^

DNA tetrahedrons are 3D DNA nanostructures made by self‐assembly of four DNA oligonucleotides. They have been considered for drug delivery applications due to their high stability and high potential for modification with targeting ligands. These structures are stable owing to the DNA strands’ complementary base pairing. This stability confirms that this nanostructure can keep its integrity under physiological conditions, permitting high‐efficacy drug encapsulation.^[^ ^51^ ^]^ Besides, DNA nanoribbons show great stability and rigidity according to the accurate folding of DNA strands into a ribbon‐like shape. They also have a large surface area that can be modified with different biomolecules, such as drugs, targeting ligands, and aptamers. This high surface area allows great drug loading capacity and the combination of multiple components within the nanostructures, principal to increased therapeutic efficiency in drug delivery applications.^[^ ^52^ ^]^

The four arms of the DNA cruciform offer multiple binding areas for drugs, allowing great drug‐loading capacity inside the DNA nanostructures. For example, some drugs, such as doxorubicin (DOX) can connect to cruciform nanostructure, since it intercalates among the DNA base pairs. The complementary regions were planned in a way that provided great amounts of Cytosine (C) and Guanine (G) bases, leading to more loading of the drug.^[^ ^53^ ^]^ On the other hand, both DNA nanocockleburs and DNA nanocentipedes show high stability and structural rigidity riding on their intricate 3D structures,  large surface area for drug binding, and a high potential for response to external stimuli, such as specific molecules.^[^ ^54^ ^]^ Overall, the advantages of different shapes of DNA nanostructures make them appropriate for targeted drug delivery applications, contributing a hopeful assay to enhance the efficacy and safety of therapeutic interventions.

**Figure 1:**
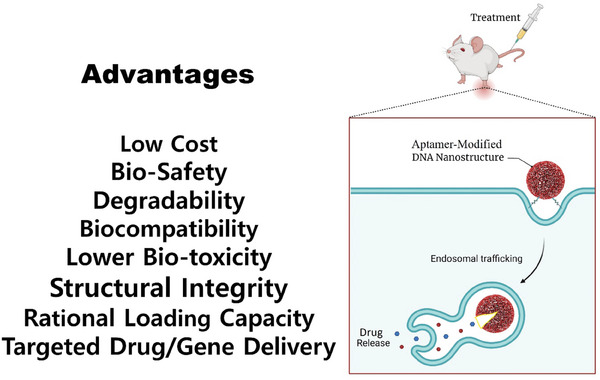
The advantages of diverse types of aptamer‐modified DNA nanostructures in targeted drug/gene delivery. Created with BioRender.com, and authors acknowledge their professional services.

The investigation into whether DNA structures can adequately penetrate cellular membranes reveals a discernible reliance on external factors or target ligands to enhance absorption. DNA nanostructures, with their inherent adaptability, allow for the strategic attachment of various ligands, including aptamers and biomolecules, thereby amplifying cellular uptake through the mediation of scavenger receptors.^[^ ^55^ ^]^ Noteworthy is the versatility offered by DNA nanostructures, permitting the incorporation of diverse ligands on a singular platform, a feature substantiated by research indicating that alterations in ligand geometry significantly impact binding affinity to specific targets. Aptamers,^[^ ^56^ ^]^ obtained through rigorous in vitro selection processes such as selective evolution of ligands by exponential enrichment (SELEX), exhibit a remarkable capacity for target specificity.^[^ ^10^, ^57^ ^]^ They facilitate drug accumulation at predetermined sites and facilitate intracellular drug delivery through receptor‐mediated endocytosis.^[^ ^58^ ^]^ Additionally, certain DNA sequences can function as direct therapeutic agents in gene therapy applications, underscoring the substantial potential of DNA in efficacious drug delivery.^[^ ^59^ ^]^ The application of AS1411 on nanosystems, serving to enhance targeted cellular uptake while safeguarding against degradation, stands out as a noteworthy example.^[^ ^60^ ^]^ Furthermore, aptamers like Muc‐1 and sg8 find effective utilization on DNA nanostructures in the context of cancer‐targeted therapy, enabling precise localization in targeted regions.^[^ ^61^ ^]^ The strategic incorporation of AS1411 onto a tubular DNA origami, particularly when affixed to endothelial tumor cells, results in the revelation of a concealed thrombin protein within its cavity.^[^ ^62^ ^]^ As a matter of example, AS1411 aptamer included multi‐story DNA nanostructure has been utilized as a DOX carrier for targeting 4T1 and MCF‐7 cell lines. The fluorometric and Gel retardation analysis assessed the DOX loading and DNA nanostructure construction. The DOX‐loaded carrier demonstrated considerable damage on nucleolin‐positive cells but nucleolin‐negative cells were intact. The rationally designed nanostructure has efficaciously internalized into targeted cells. The tumor growth has been restricted through considerable accumulation in the tumor.^[^ ^63^ ^]^ **Figure** **1** visually articulates various DNA nanostructures modified with aptamers, elucidating their advantages in the realm of targeted drug delivery.

## 2 DNA Nanostructures for Aptamer‐Based Targeted Delivery

### 2.1 DNA Origami Nanostructures

**Figure 2:**
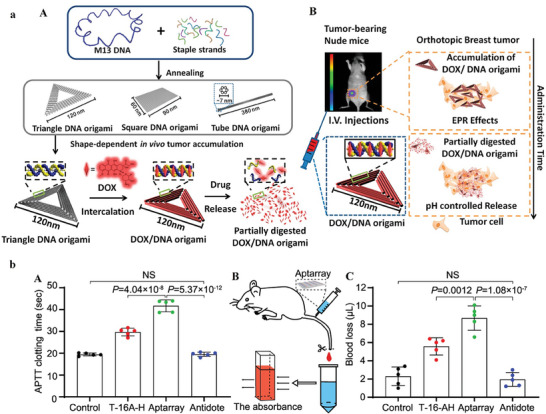
a) The diagram explicates the conceptual framework of the DNA carrier–drug complex. In Panel (A), an elongated single‐stranded DNA scaffold (depicted in blue as M13mp18 phage genomic DNA) engages in hybridization with intentionally designed auxiliary strands, resulting in the formation of triangular, square, and tubular origami structures. Biodistribution studies encompass both unstructured M13 DNA and diverse DNA origami nanostructures within a subcutaneous breast tumor model. Following in vivo biodistribution, the triangular DNA origami demonstrates optimal tumor accumulation and is subsequently utilized for doxorubicin intercalation (illustrated in red). The Watson–Crick base pairs with DNA origami's double helices act as docking sites of doxorubicin intercalation. In Panel (B), DOX/DNA origami complexes, administered via tail injection, traverse the bloodstream, accumulating in breast tumors of nude mice due to enhanced permeability and retention (EPR) effects. Reproduced with permission from.^[^ ^65^ ^]^ Copyright 2014, American Chemical Society. b) Panel (A) illustrates the treatment of mice (*n*  =  5) with buffer (Control), Aptarray (≈560 nM, 100 µL), or T‐16A‐H (20 µM, 100 µL), through a singular tail vein injection. Subsequent intravenous administration of antidotes, followed by Aptarray, is conducted for in vivo neutralization, with quantification of plasma APTT levels. Panel (B) presents a schematic representation of a murine tail‐transection bleeding model. In Panels (A and C), mice (*n*  =  5) receive treatment with buffer (Control), Aptarray (≈560 nM, 100 µL), or T‐16A‐H (20 µM, 100 µL), and antidotes are administered to anticoagulated animals for neutralization. Tail tip amputation facilitates the measurement of blood loss. The data, reflecting the mean ± standard deviation from five independent replicates, undergo statistical analysis using one‐way ANOVA with the Tukey post hoc test (NS, *p * >  0.05). Reproduced with permission from.^[^ ^68^ ^]^ Copyright 2021, Nature Communication.

DNA origami, exhibiting diverse sizes and morphologies, functions as a versatile carrier for transporting biomolecules, including DOX, various proteins, and therapeutic nucleic acids, thereby facilitating their efficient intracellular delivery.^[^ ^7a^ ^]^ These nanostructures demonstrate distinct responses to environmental stimuli, making them well‐suited for controlled drug and gene delivery systems.^[^ ^64^ ^]^ Among the critical determinants influencing their efficacy, the configuration of DNA origami emerges as a paramount factor. Recent investigations emphasize the substantial role played by nanostructure shape in drug delivery. Remarkably, the application of an open‐caged pyramidal DNA nanostructure has yielded a substantial improvement in the targeted delivery of DOX to breast and liver cancer cells, surpassing the efficacy observed with free DOX. Furthermore, triangular DNA origami structures have exhibited a distinct propensity for enhanced accumulation at tumor sites, demonstrating superior efficacy compared to structures characterized by rectangular or rod‐shaped configurations (**Figure** **2**a).^[^ ^65^ ^]^ Both DNA origami and aptamers, both comprised of nucleic acids, demonstrate a notable biocompatibility, wherein aptamers exhibit the capacity to establish connections with sequences through base pairing. The precise arrangement of aptamers on DNA origami is achievable with considerable accuracy, facilitated by the close juxtaposition of DNA helices within these structural configurations. Additionally, DNA origami, secured by strand‐dislocation locks, offers a foundational framework for the substitution of these locks with aptamers. This presents a novel avenue for the implementation of a ligand‐binding‐mediated unlocking mechanism.^[^ ^66^ ^]^ Wu and co‐workers developed a precision‐guided‐missile‐like DNA nanostructure for recognizing cancer cells. This DNA structure was comprised of a head for loading the drug and a control/guidance for recognizing cancer cells. The warhead was a 3D DNA structure assembled from other DNA sequences. Using this structure, the specific detection was done by the disassembly that was facilitated via the aptamers, allowing the drug delivery. This DNA nanostructure has high stability according to the intrinsic DNA structure. The DNA structure showed high promise for use in precisely targeted therapy and drug delivery.^[^ ^67^ ^]^ DNA origami‐based structures, designed to impede thrombin activity and thrombus formation, have exhibited notable anticoagulant efficacy. Through the incorporation of thrombin‐binding aptamers, these nanoarchitectures have demonstrated robust recognition and inhibition of thrombin across diverse biological settings, including human plasma, fresh whole blood, and murine models. The in vivo evaluation of the Aptarray using Plasma clotting time (APTT) illustrates a substantial prolongation in clotting time post‐administration. Mice subjected to Aptarray treatment manifest prolonged clotting times (41.7 ± 2.7 s), surpassing those of the buffer (19.4 ± 0.7 s) and T‐16A‐H treated groups (29.7 ± 1.8 s) (Figure 2b).^[^ ^68^ ^]^

**Figure 3:**
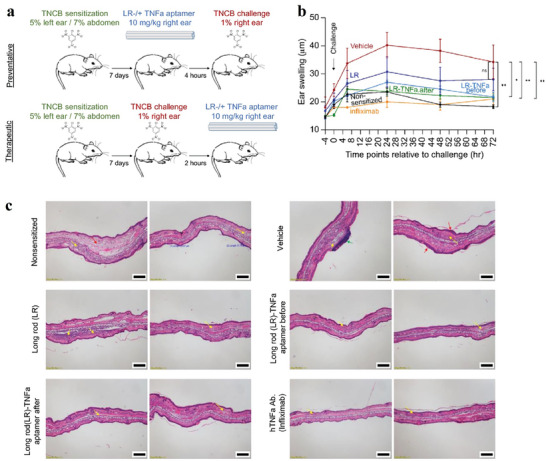
a) The figure depicts the ameliorative impact of TNFa aptamers coated DNA origami (long rod) on inflammation indices in a DTHR mouse model. The figure outlines TNCB‐induced DTHR in humanized TNFa mice under preventative and therapeutic treatment regimens. b) measurements of ear thickness before and at specified time intervals after the TNCB challenge are presented for different mouse cohorts. Administration of Long rod − / + TNFa aptamer and infliximab occurs either before or after the challenge. The data, representing the two independent experiments’ mean values ± SEM (*n*  =  4), undergo considerable determination through ANOVA with Tukey's multiple comparing test (^*^ *p * <  0.03; ^**^ *p * < 0.008; ns means: not significant). c) ear sections’ histopathological images from designated mouse groups, illustrating tissue alterations and inflammatory responses. All scale bars are 200 µm. Reproduced with permission from.^[^ ^69^ ^]^ Copyright 2023, Scientific reports.

In a study involving humanized mice sensitized to TNF‐alpha and challenged with TNCB‐induced inflammation, diverse treatments were administered. These treatments included elongated structures coated with PEG‐polylysine but lacking aptamers, elongated structures containing 20 TNF‐alpha aptamers (referred to as long rod‐TNFa aptamer), and infliximab. The investigators demonstrated that when DNA was coupled with polyethylene glycol (PEG), it maintained structural integrity postinjection (**Figure** **3**). Consequently, the aptamers targeting TNF‐alpha effectively mitigated inflammatory responses in mice humanized for TNF‐alpha. This innovative approach provides a programmable alternative to monoclonal antibodies, facilitating spatial control of drug activity.^[^ ^69^ ^]^

**Figure 4:**
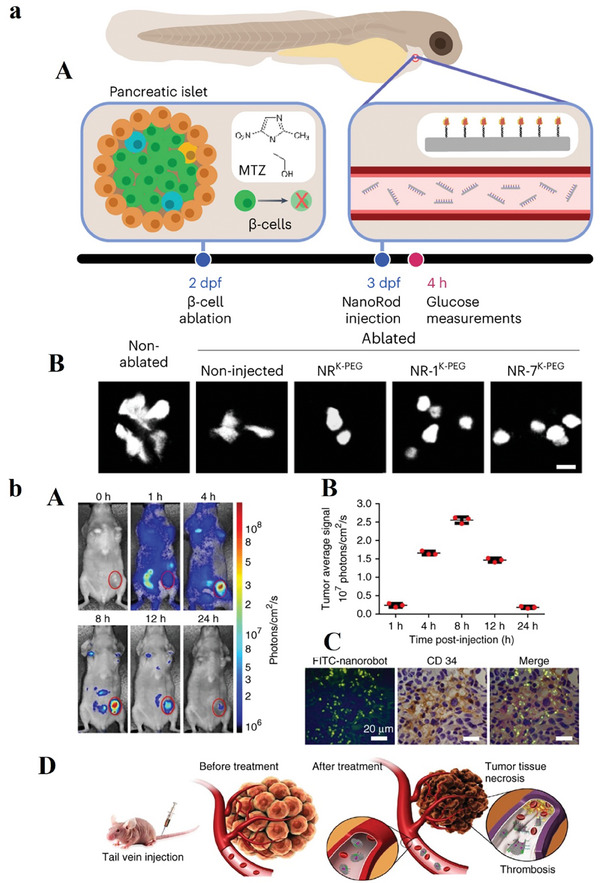
a) (A) The depiction provides the zebrafish model's overview, wherein the enzyme nitro‐reductase (NTR) is expressed under the insulin promoter regulatory control. This regulatory arrangement facilitates the conversion of the MTZ compound to a cytotoxic component, ultimately resulting in the ablation of β‐cells. Larvae underwent MTZ treatment at 2 dpf for a duration of a day. Double‐transgenic larvae, denoted as Tg(ins: CFP‐NTR); Tg(ins: Kaede), were employed for the visualization of β‐cells using the fluorescent protein Kaede. Intravenous injections of NRK‐PEG, NR‐1K‐PEG, or NR‐7K‐PEG were administered at 3 dpf, and the levels of free glucose were subsequently measured at 4 h postinjection. (B) The utilization of confocal microscopy has been employed to capture the expression of the Kaede fluorescent protein within pancreatic β‐cells under the specified experimental conditions. The scale bar corresponds to 10 µm. Reproduced with permission from.^[^ ^70^ ^]^ Copyright 2023, Nature Nanotechnology. b) (A) Optical imaging was conducted on a mouse hosting a breast tumor of a human (MDA‐MB‐231) before and after the intravenous introduction of Cy5.5‐labeled nanorobots. A discernible intense fluorescent signal was exclusively detected in the tumor site 8 h postinjection. The 0 h time point designates the pre‐injection state. The presented images demonstrate the 3 experiments. (B) Quantification of in vivo fluorescence intensity of tumor region at designated time intervals following nanorobot administration. Error bars show the mean ± standard deviation of 3 experiments. (C) FITC‐labeled nanorobots intravenous injection into mice with tumors (MDA‐MB‐231). Tumors have been harvested 8 h postinjection, and subsequent staining with an anti‐CD34 antibody enabled examination through confocal microscopy. The nanorobot (green) is observed in regions rich in blood vessels (anti‐CD34; brown). Nuclei are denoted in blue. The presented images show 3 different experiments, with scale bars set at 20 µm. (D) illustration elucidates the nanorobot‐Th therapeutic mechanism within tumor vessels. DNA nanorobot‐Th administration via tail vein injection to breast tumor xenografted mice targets tumor‐associated vessels, delivering thrombin. The nanorobot‐Th, recognizing nucleolin, binds to vascular endothelium, subsequently opening to expose encapsulated thrombin, inducing localized thromboses, tumor infarction, and cell necrosis. Reproduced with permission from.^[^ ^71^ ^]^ Copyright 2018, Nature Biotechnology.

In the context of an in vivo zebrafish model simulating diabetes, the study conducted a comparative analysis of the impacts of multivalent (NR‐7) and monovalent (NR‐1) insulin administration (**Figure** **4**a).^[^ ^70^ ^]^ Rod‐shaped nanostructures were developed through the combination of insulin and DNA origami, featuring diverse insulin valences and precisely defined spacings. The results revealed that an increase in insulin valency per nanostructure resulted in a prolonged duration of these constructs at insulin receptors. Both insulin valency and spacing were identified as significant factors influencing the levels of activation exhibited by the insulin receptor in adipocytes. A platform of autonomous DNA nanorobots was developed to facilitate precise drug delivery to tumors. Constructed through DNA origami, the nanorobot features an external layer incorporating a DNA aptamer that specifically targets nucleolin, a protein expressed on endothelial cells associated with tumors. Internally, the nanorobot encapsulates thrombin, a protease involved in blood coagulation. In vivo experiments involving mice with orthotopic tumors revealed a notable efficacy of the nanorobot, demonstrating a sevenfold increase in accumulation within tumors compared to non‐targeted nanotubes (Figure 4b). Safety evaluations in both mice and Bama miniature pigs established its immunological inertness. In addition, the results showed nanorobot‐induced intravascular thrombosis at the tumor site, leading to tumor necrosis and impeding growth.^[^ ^71^ ^]^

Aptamer‐modified DNA origami emerges as a pivotal platform for addressing drug resistance within the realm of academic inquiry. Employing nanocarriers constructed from DNA origami offers a sophisticated means for concurrently delivering DOX and various antisense components, such as antisense oligonucleotides (ASOs), B‐cell lymphoma 2 (Bcl2), and P‐glycoprotein (P‐gp), into cancer cells, thereby amplifying the effectiveness of cancer therapy. The regulated release of DOX in acidic pH environments and the targeted delivery of ASOs, prompted by glutathione from the nanocarriers, constitute integral facets of this methodology. Aptamer‐DOA notably enhances therapeutic efficacy in MCF‐7/ADR and HeLa cells. This auspicious nanocarrier embodies a pioneering approach for efficacious intervention in drug‐resistant cancers within the academic discourse.^[^ ^72^ ^]^ However, effective delivery of therapeutic drugs remains a main challenge. DNA origami is enabled by penetrating membranes, at that time accumulating in tumor tissue by the increased permeability. Besides, AS1411 aptamer improves internalization by nucleolin‐facilitated pinocytosis.^[^ ^73^ ^]^ Taghdisi et. al. designed an active targeting DNA nanostructure (3‐way junction pocket) as a nanocarrier of DOX. The 3‐way junction pocket has been composed of AS1411 aptamer (three strands) as nucleolin target and therapeutic aptamer which demonstrated pH responsiveness and considerable serum stability. The 3‐way junction pocket DNA nanostructure which is loaded with DOX considerably lessens the cytotoxicity of DOX against the cells that did not target (targeted cells: 4T1 (breast cancer) and PC‐3 (prostate cancer)).^[^ ^74^ ^]^

**Figure 5:**
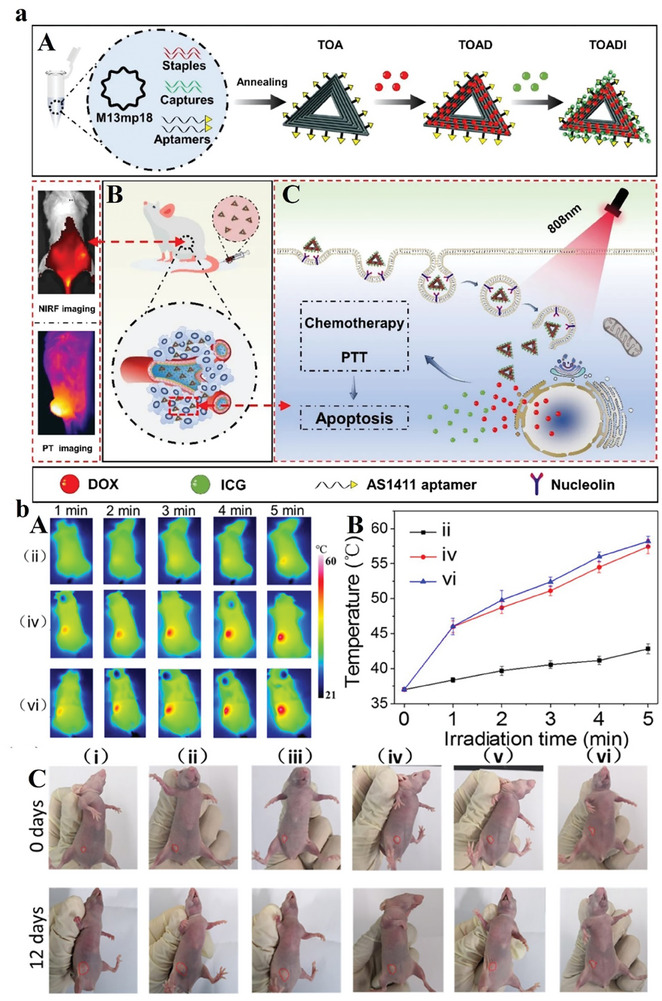
a) Illustrative representation of the DNA origami‐based system designed for cancer, encompassing therapeutic interventions against breast cancer. Reproduced with permission from.^[^ ^75^ ^]^ Copyright 2023, Journal of Nanobiotechnology. b) In vivo synergistic chemophotothermal therapy. (A) Thermal photo capturing and (B) tumors’ temperature profiles after the saline injection, DNA‐modified AuNPs, or nano‐agent plus irradiation. (C) the mice digital micrographs. Reproduced with permission from.^[^ ^79^ ^]^ Copyright 2019, American Chemical Society.

An AS1411‐modified DNA origami (TOA) can be applied for the co‐delivery of DOX and a photosensitizer (indocyanine green, ICG). This AS1411‐based platform as an aptamer of nucleolin powerfully improves the carrier's endocytosis more than three folds through tumor cells. These nanocarriers can controllably deliver the DOX into the nucleus by the photothermal effect of ICG via NIR, and the acidic condition of lysosomes. With the synergetic effect of DOX and ICG, the origami platform displays an important therapeutic effect of ≈90% prevention of tumor growth with high toxicity. With these outstanding benefits, this biocompatible DNA origami platform has good potential to load oligonucleotides, and drugs for cancer therapy (**Figure** **5**a).^[^ ^75^ ^]^ Generally, one of the important roles of DNA origami structures, especially in MDR, is the co‐delivery of multiple therapeutic components.^[^ ^76^ ^]^ Development of a multifunctional DNA origami platform via the mixture of DOX and GNRs t can be used to downregulate the expression of Pgp and avoid drug resistance of MCF7/ADR cells in vitro. In fact, with this platform, we attain efficient cellular uptake and combined chemotherapeutic and photothermal therapy for cancer therapy.

Earlier studies showed that triangular DNA origami has a key role in DOX delivery. Lately, scientists have established that the tubular‐shaped DNA origami structure can be applied as a carrier for gene/ drug delivery.^[^ ^77^ ^]^ Several studies have shown that the internalization of DNA origami structures relies on the type of cell line, size, and shape. DNA origami structures with a size of 50 to 80 nm are good for internalization. Additionally, an array of DNA origami structures showcases significant efficacy in the encapsulation and delivery of pharmaceutical agents. Extensive investigations have underscored the pivotal role of DNA origami nanocarrier dimensions in influencing diverse properties, particularly those integral to drug delivery systems. Upon meticulous examination of the Donut, Disc, and Sphere origami nanostructures, it became apparent that the Donut conformation, characterized by its heightened stability, demonstrated commendable proficiency in accommodating doxorubicin (DOX) with a notable capacity. Moreover, the study revealed that the presence of MUC1 not only enhanced cellular uptake within MCF‐7 cell lines but also exhibited heightened cytotoxicity against cancer cells.^[^ ^78^ ^]^ In experimental scenarios involving murine subjects bearing HeLa tumors, the nano‐agent demonstrated promising potential for efficient chemo‐photothermal synergy. This resulted in the eradication of tumors while concurrently mitigating undesired side effects.^[^ ^79^ ^]^ According to previous studies, the nanosphere increased the uptake of DOX within the cell via intrusive efflux procedures. In HaCaT cells, the DOX‐aptamer‐sphere exhibited improved toxicity by 10% of DOX concentration, which occurred due to the poor response to DOX treatment in MCF‐7 cells.^[^ ^80^ ^]^ Nanosphere DNA origami not only increases therapeutic efficacy, but they along with aptamers could decrease the side effects of drugs. A smart nano agent system was developed using a DNA complex to modify gold nanoparticles, enabling efficient transport of the chemotherapeutic drug DOX to tumor sites. This approach facilitated the effective delivery of the chemotherapeutic DOX to malignant sites. The nano‐agent exhibited swift drug release within acidic tumor cells, enabling intracellular ATP imaging and demonstrating notable in vivo photothermal capabilities (Figure 5b).

### 2.2 DNA Tetrahedron Nanostructures

Tetrahedral DNA structure (TDNs) is a 3D nanostructure fabricated by the complementary pairing of four ssDNA. This nanostructure has been developed as a hopeful delivery platform according to its great stability, compatibility, and rich functional modification areas, appropriateness for various drug uptake rates.^[^ ^81^ ^]^ The use of TDNs and biomolecules has been developed, which resolves the significant challenges in the progress of drugs, including poor membrane permeability, no targeting activity, and instability.^[^ ^82^ ^]^ Besides, TDNs are powerfully internalized via cells even in the transfection agents’ absence. Tetrahedral framework nucleic acids (tFNAs) exhibited low toxicity, high with a simple synthesis method, which formed through four single DNA strands. They are more stable compared to the single strands and can simply enter the cell membrane through caveolin‐mediated endocytosis. Besides, tFNAs have multiple modification areas, and therapeutic oligonucleotides, which can be loaded into tFNAs. Thus, tFNAs are estimated to become an appropriate nanocarrier for the delivery of siRNA.^[^ ^83^ ^]^ siRNA is established to successfully knock‐down the target gene in cells, which is deliberated a hopeful approach for gene therapy.^[^ ^84^ ^]^

**Figure 6:**
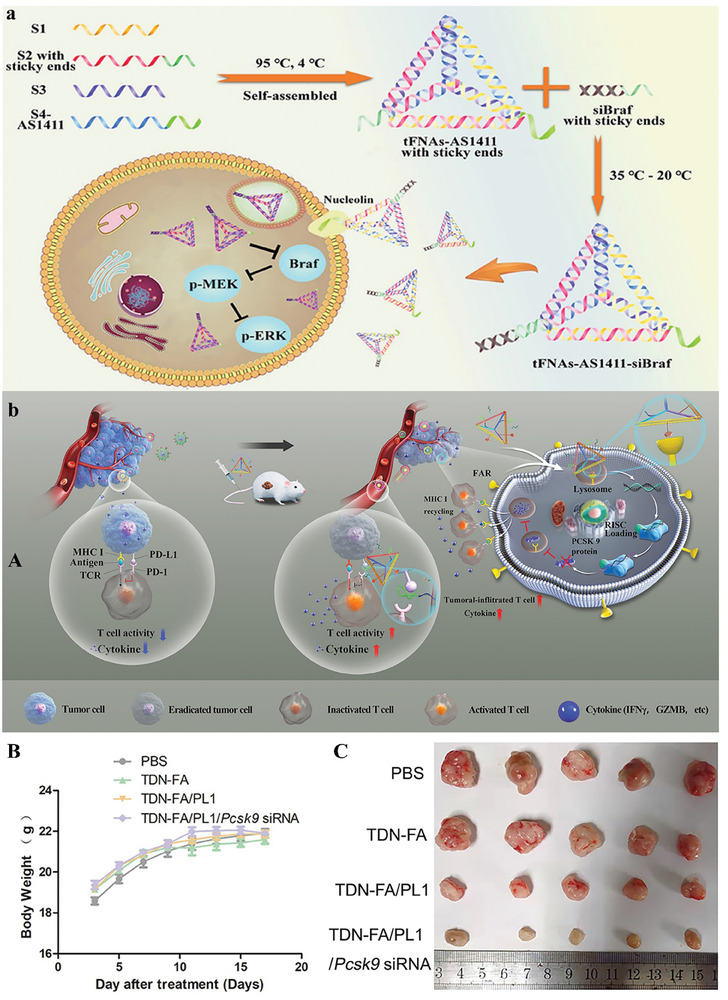
a) Schematic illustration of the tetrahedral structure loaded with AS1411 in order to release siRNA in malignant melanoma. Reproduced with permission from.^[^ ^85^ ^]^ Copyright 2021, American Chemical Society. b) (A) Antitumor therapy of TDN‐FA/PL1/Pcsk9 siRNA via nanoplatform separated, delivered the Pcsk9 siRNA in tumor, and decreasing PCSK9‐mediated MHC I degradation. (B) Body weight of mouse. (C) different mice tumors photo. Reproduced with permission from.^[^ ^86^ ^]^ Copyright 2022, American Chemical Society.

tFNAs‐AS1411‐siBraf formed by a simple method with great yield can be used for siRNA delivery. Here, with the help of tFNAs, siBraf can be entered by the cell membrane. Moreover, the cellular uptake and gene silencing capability of this carrier were increased by the modification of AS1411 aptamer (**Figure** **6**a). Nevertheless, after the entry into cells, tFNAs and AS1411 display no sign of interfering via siBraf. The incorporation of AS1411 aptamer into DNA nanostructure can help tFNAs to increase the cellular uptake efficiency of siBraf. This platform joined with tFNAs can be used as a high‐promise carrier for gene delivery. The approach of joining siRNA with tFNA nanocarriers and targeting aptamers could be a useful platform for different gene delivery systems.^[^ ^85^ ^]^ TDNs showed multi‐functional potential in the treatment of colorectal cancer. Researchers developed an assay to “backpack” aptamer PL1, which connects to Pcsk9 siRNA and PD‐L1 on TDNs via DNA hybridization. Besides, this structure can connect to folic acid receptors which provide a targeted delivery system. The TDN exhibited the capacity to mobilize immune cells with precision toward targeting colorectal cancer cells. This resulted in a substantial reduction of 83.4% in the growth of tumor tissues in murine models of colorectal cancer after ten intravenous administrations, devoid of any observable toxicity. Notably, the cancer‐targeting functionality of the TDN, guided by folic acid, facilitated the specific delivery of TDN‐Pcsk9‐siRNA into cancer cells. This targeted delivery mechanism played a pivotal role in disrupting the interaction between programmed cell death protein 1 (PD‐1) and programmed death ligand 1 (PD‐L1). The discernible outcome was a 1.6‐fold enhancement in T cell activity. Concurrently, the introduction of small interfering RNA (siRNA) led to a reduction in Pcsk9 expression by 65%, concomitant with the facilitation of intertumoral infiltration by T cells. This nuanced strategy not only demonstrated the efficacy of the TDN but also highlighted its potential for immune modulation and precision‐targeted therapy in the context of colorectal cancer (Figure 6b).^[^ ^86^ ^]^

The data obtained from the study reveals the efficacy and safety of the multifunctional structure in the context of colorectal cancer therapy, thereby opening up the window of opportunity for the utilization of DNA nanotechnology in cancer research. Lin and colleagues have spearheaded the development of a delivery system based on Tetrahedral DNA nanostructures, designed to facilitate the transportation of antisense Peptide Nucleic Acid (PNA) and aptamers into cellular environments. The TDN‐based delivery systems undergo application in four distinct modification assays, encompassing the utilization of antisense, drug incubation, and a specialized coating agent. Notably, TDNs exhibit a diverse range of properties essential for targeted therapy, leading to a substantial enhancement in cellular uptake.^[^ ^87^ ^]^

**Figure 7:**
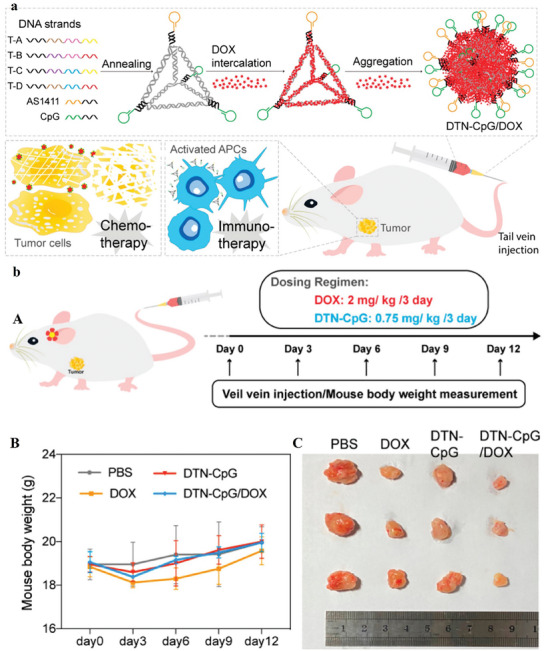
a) Elaboration of the Synthesis Procedure for DTN‐CpG/DOX Nanoparticles and Their Application in the Context of Synergistic Cancer Therapy. b) Dosing routine for antitumor study. (B) Mice body weight in various groups. (C) Representative photographs of eliminated tumors in various groups on the 15th day. Reproduced with permission from.^[^ ^89^ ^]^ Copyright 2022, American Chemical Society.

The utilization of DNA tetrahedra as a drug delivery platform for the administration of the anticancer agent DOX presents several noteworthy advantages. This molecular architecture can autonomously self‐assemble from various ssDNA components, resulting in the creation of a structurally stable entity characterized by meticulously controlled dimensions. The inherent nature of these nanostructures allows for the effective encapsulation of pharmaceutical agents within their molecular strands, thereby facilitating the delivery of a heightened quantity of drugs. Moreover, the strategic integration of a tumor‐targeting aptamer with the DNA tetrahedron follows the principles of base pairing in a self‐assembled fashion. This innovative approach obviates the requirement for catalyst‐mediated chemical reactions, consequently eliminating the need for purification protocols and concurrently mitigating the associated synthesis costs.^[^ ^88^ ^]^ DNA tetrahedra was utilized to construct co‐delivery nanoplatforms amalgamating the chemotherapeutic agent doxorubicin and the immunotherapeutic CpG oligodeoxynucleotides. The resultant DTN‐CpG/DOX nanoparticles manifest synergistic therapeutic effects characterized by heightened immunostimulatory activity and conspicuous antitumor efficacy. Consequently, researchers incorporated a drug delivery system (DDS) to facilitate synergistic cancer therapy, utilizing DNA nanostructures that incorporate the immunotherapeutic CpG oligodeoxynucleotides (CpG) and the chemotherapeutic agent DOX (**Figure** **7**a). The findings demonstrate that the adapted DTN, when equipped with the AS1411 aptamer, effectively targets tumor sites. Subsequently, DOX eradicates tumor cells upon reaching the designated site, leading to the liberation of tumor‐associated antigens.^[^ ^89^ ^]^ The aptamer‐modified DNA tetrahedron shows greatly targeted cellular uptake with the alteration of only one site. The aptamer can be prepared exactly on the summit of a self‐assembled DNA tetrahedron through hybridization. After loading the DOX, the aptamer cluster‐based platform stimulates selective prevention of tumor cell proliferation through targeted delivery. This DNA nanostructure offers a new method for the progress of targeted delivery.^[^ ^90^ ^]^

The intracellular distribution of AS1411‐modified TDNs, specifically Apt‐TDNs, was methodically scrutinized in comparison to conventional TDNs across diverse cell types experiencing hypoxic conditions. Additionally, the impact of Apt‐TDNs and TDNs on cellular proliferation and the cell cycle under hypoxic circumstances was thoroughly explored. Within this intricate cellular milieu, a noteworthy accumulation of Apt‐TDNs within the nucleus of MCF‐7 cells was evident, surpassing the relative entry into L929 cells. Remarkably, the influx of TDNs into MCF‐7 cells fell notably short compared to the robust penetration observed with Apt‐TDNs. Noteworthy outcomes emerged as Apt‐TDNs demonstrated the capacity to impede the growth of MCF‐7 cells while concurrently fostering the growth of L929 cells. In contrast, TDNs exhibited a growth‐promoting effect on both MCF‐7 and L929 cells.^[^ ^91^ ^]^ Platinum‐based pharmaceutical agents have attained widespread utilization as primary chemotherapeutic modalities in contemporary clinical practice, addressing a spectrum of cancerous conditions. A seminal contribution by Ding et al. involves the innovative design of a DNA nanoplatforms explicitly engineered for the targeted delivery of platinum drugs. The foundational architecture of this groundbreaking platform relies on the implementation of a double‐bundle DNA tetrahedron. The integration of the platinum‐based drug (56MESS) into the DNA double helix is executed with precision through the mechanism of intercalation, facilitating its incorporation onto the double‐stranded DNA tetrahedron. To realize exacting delivery, an anti‐EGFR nanobody is meticulously attached to the DNA, forming an intricate assembly on the double‐stranded DNA tetrahedron. The investigative findings underscore the exceptional selectivity of this engineered DNA nanoplatform, particularly in its capacity to target cells overexpressing EGFR. The outcome of such targeted delivery manifests in potent antitumor activity, concurrently mitigating discernible systemic side effects. This sophisticated DNA nanoplatform represents a noteworthy advancement in the pursuit of effective and targeted cancer therapeutics.^[^ ^92^ ^]^

Within the domain of liver‐specific microRNAs, microRNA‐122 (miR‐122) assumes a central role in steering the differentiation of stem cells into hepatocytes.^[^ ^93^ ^]^ The TDN nanoplatform, distinguished by its structural characteristics, exhibits considerable potential for inducing human mesenchymal stem cells (hMSCs) to differentiate into hepatocyte‐like cells (HLCs). This process hinges on the efficient transfer of liver‐specific miR‐122 to hMSCs, independent of extrinsic factors. In contrast to miR‐122 alone, the miR‐122‐functionalized TDN (TDN‐miR‐122) significantly enhances protein expression levels linked to mature hepatocyte markers and hepatocyte‐specific genes in hMSCs. This underscores the proficiency of TDN‐miR‐122 in activating hepatocyte‐specific traits within hMSCs, thus bolstering the viability of in vitro cell‐based therapeutic strategies.^[^ ^94^ ^]^ The transcriptomic analysis further unveils the potential mechanisms through which TDN‐miR‐122 facilitates the differentiation of hMSCs into functional HLCs. The TDN‐miR‐122‐altered hMSCs display a distinctive hepatic cell morphology phenotype, accompanied by a noteworthy up‐regulation of specific hepatocyte genes and augmented hepatic biofunctions compared to undifferentiated MSCs.^[^ ^91^ ^]^ Preclinical in vivo transplantation studies affirm the effectiveness of TDN‐miR‐122‐altered hMSCs, whether administered independently or in conjunction with TDN, in mitigating acute liver failure injury. This therapeutic efficacy arises from the enhancement of hepatocyte functions, anti‐apoptotic interventions, the promotion of cellular proliferation, and the induction of anti‐inflammatory responses.

The MUC1 protein represents a significant target in cancer therapy due to its prevalent overexpression across various cancer types. In a thorough investigation, a drug delivery system centered around MUC1 was meticulously devised, utilizing an aptamer with the capacity to selectively recognize MUC1 and a DNA nanostructure designed for the precise delivery of drugs within its DNA matrix. This intricately designed platform, characterized by a diameter of 12 nm, adeptly establishes connections with MUC1‐positive MCF‐7 cancer cells. A detailed examination of drug loading capabilities revealed that the Apt‐Td construct facilitated the delivery of 25 drug molecules. Furthermore, this platform not only efficiently transported drugs into the target cells but also demonstrated a discernible reduction in DOX uptake by MUC1‐negative control cells. Remarkably, the platform exhibited an enhanced cytotoxic effect against MUC1‐positive cancer cells compared to normal cells. Cell viability assessments for DOX and Apt‐Td‐DOX in MCF‐7 cells resulted in percentages of 44% and 35%, respectively. Conversely, MDA‐MB‐231 cells exhibited lower toxicity, registering percentages of 47% and 86%. These compelling outcomes suggest that Apt‐Td holds considerable promise as a nanoparticle platform for advancing breast cancer treatment.^[^ ^88b^ ^]^ Furthermore, investigators proposed an innovative strategy involving 1–3 MUC1‐aptamer‐modified DNA tetrahedra for DOX delivery in breast cancer. Their investigations underscored the pivotal role of the number of aptamers on the DNA tetrahedron, revealing a substantial influence on cellular uptake efficacy. Notably, the aptamers markedly enhanced uptake efficacy in tumor cells while concurrently reducing uptake efficiency in normal cells. This novel approach contributes valuable insights into the potential optimization of targeted drug delivery systems for enhanced therapeutic outcomes within an academic context.^[^ ^96^ ^]^

Photosensitizers are significant for photodynamic therapy; although, common photosensitizers show low selectivity and a big challenge for their release. The study aims to produce a targeted delivery platform for novel photosensitizers by aptamer‐modified DNA tetrahedra. The TMPyP4 as a photosensitizer, can be loaded into TDNs according to the great affinity of TMPyP4 for DNA. This platform permitted the targeted delivery of TMPyP4 to tumors. Additionally, MTT increases the construction of ROS along with toxicity in cells, although less killing influence was detected in cells. With this study we can plan a targeted delivery system of photosensitizers based on DNA nanostructure, therefore providing an assay for the targeted delivery of photosensitizers.^[^ ^97^ ^]^

The refinement of selectivity within delivery systems can be achieved through the integration of dual or multiple targeting mechanisms. It is of paramount importance to meticulously develop carriers featuring a dual “key‐lock” design tailored explicitly for drug delivery, with the primary aim of mitigating undesirable side effects.^[^ ^98^ ^]^ In pursuit of this objective, researchers have devised a sophisticated drug delivery platform by modifying DNA tetrahedrons with MUC1 and AS1411 aptamers. This strategic modification has resulted in a noteworthy enhancement in the specific uptake of DOX and its overall therapeutic efficacy.

This intricate platform encompasses three fundamental components: first, a DNA tetrahedron core designed for ligand conjugation and DOX loading; second, the incorporation of the MUC1 aptamer probe; and third, the inclusion of the AS1411 aptamer, strategically hybridized to extensions on three apexes for connection to nucleolin. The initial targeting process involves the MUC1 aptamer, which selectively homes in on MUC1 present on the cell surface. This targeting event triggers a reorganization of the aptamer, leading to the delivery of a complementary sequence with a quencher leads to fluorescence recovery. Upon internalization into cells, the aptamer establishes a connection with nucleolin, facilitating the intracellular delivery of DOX into the nucleus. Remarkably, the MUC1‐Td‐AS1411‐DOX nanoplatforms demonstrate reduced toxicity toward MUC1‐negative HL‐7702 cells (*p* < 0.01) compared to its impact on MCF‐7 cells. Furthermore, it exhibits enhanced efficacy against DOX‐resistant MCF‐7 cells. Consequently, this nanoplatform emerges as a promising and potentially transformative approach for cancer therapy.^[^ ^98^ ^]^

**Figure 8:**
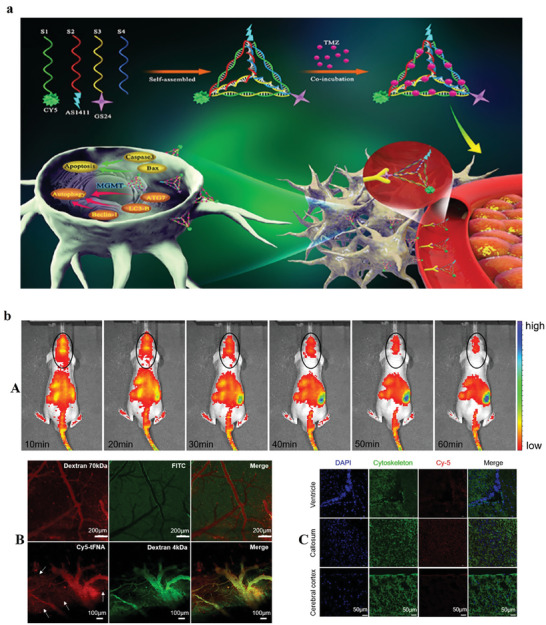
a) Schematic illustration of prepared nanostructure and its utilization. b) (A) In vivo experiments of injecting Cy5‐tFNA into BALB/c nude mice. (B) Presence of dextran 70 kDa in vascular lumen (C) Distribution of tFNA in the various brain parts such as ventricle, callosum, and cerebral cortex, scale label = 50 µm. Reproduced with permission from.^[^ ^95^ ^]^ Copyright 2019, American Chemical Society.

Temozolomide (TMZ) is an alkylating agent that enters the blood‐brain barrier (BBB) and is used for glioblastoma. Tetrahedral framework nucleic acid (tFNA) is widely used in biomedical applications because of its biosafety and biocompatibility. tFNA also displays anti‐inflammatory, and neuroprotective effects. Besides, tFNA could efficiently deliver drugs to increase the lethality of the cancer cell or converse the methicillin resistance. For example, researchers applied two aptamers (GS24 and AS1411) modified tFNA assembled with TMZ to produce nanoplatforms for glioblastoma therapy. The platform could enter the BBB, causing an improvement in TMZ's lethal effect on glioblastoma. In general, two advantages are obtained from these platforms: i) killed TMZ‐sensitive cells, and ii) overwhelmed TMZ‐resistance via consuming DNA‐methyltransferase.^[^ ^95^ ^]^ This nanocarrier showed that modified tFNA with GS24 aptamer can cross the BBB of mice. The results showed that tFNA could remain for 60 min in the brain vessel, proposing that tFNA could be a promising targeted delivery system (**Figure** **8**).

Gint4.T recognized as an aptamer with the ability to specifically target the platelet‐derived growth factor receptor β (PDGFRβ) on tumor cells, has been harnessed for the modification of DNA TDNs to advanced delivery systems. The TDNs underwent a self‐assembly process, resulting in a discernable size of 10 nm and a negative charge. Researchers strategically employed Gint4.T‐modified DNA tetrahedrons to facilitate the loading of DOX for the purpose of impeding glioma cell growth through targeted interaction with PDGFRβ.^[172]^ The incorporation of dual/multiple targeting mechanisms has been evidenced to enhance the precision of drug release. The DOX@Apt‐TDN demonstrated a marked increase in apoptosis rates, heightened cell cycle arrest, and elevated toxicity specifically toward U87MG cells. The essential task of overseeing tumor biomarkers assumes paramount significance in the domains of cancer diagnosis, monitoring disease progression, and evaluating the effectiveness of therapeutic interventions. However, the potential for false‐positive feedback arises when attempting to discern single or multiple biomarkers sharing identical spatial locations. To address this challenge, a DNA nanodevice was developed, characterized by its simplicity, sensitivity, and rapid functionality, for the in situ sequential imaging of multilayer biomarkers, enabling the precise identification of cancer cells. This construct integrates DNA logic gates with localized Chain Reaction (CHA), resulting in Targeted DNA Logic Nanodevices (TDLNs) that exhibit high stability, facile assembly (Figure 6a), and fine biocompatibility. Notably, TDLNs demonstrate the ability to simultaneously monitor membrane‐associated MUC1 and cytoplasmic miR‐21 within living cells, thereby enhancing their utility. Furthermore, these intelligently designed nanodevices proficiently execute AND logic operations when triggered by the concurrent stimulation of MUC1 and miR‐21, enabling accurate differentiation and identification of various cell types. Consequently, the application of these advanced nanodevices holds substantial promise in enhancing the reliability of diagnostic procedures and assessing the efficacy of therapeutic interventions.^[^ ^99^ ^]^

### 2.3 DNA Nanoribbon

Recently, efficient, and low‐cost DNA nanostructures, such as nanoribbons, have drawn a great deal of attention. DNA nanoribbons can overcome some challenges related to the DNA origami properties, such as being time‐consuming and expensive.^[^ ^100^ ^]^ DNA is dsDNA. Nanoribbon structures are formed by rolling‐circle amplification (RCA), Hybridization chain reaction (HCR), and catalytic hairpin assembly (CHA). Using these technologies, researchers created various nanoribbons to release photosensitizers, drugs, and siRNA.^[^ ^56^, ^101^ ^]^ The high uptake and delivery of drugs/genes can occur with AS1411 aptamer, which can reduce cell efflux of DOX. DNA nanoribbons have a high ability to transport high‐load drugs in target cells. Ren et al. developed a dual lock system for siRNA delivery. They planned a siRNA‐loaded nanoribbon, which has been modified with a hairpin structure to perform as a smart system. After the nanoribbon knew two locks, sgc4f, and sgc8c aptamer on the cell membrane, could it release siRNA to target cells?^[^ ^102^ ^]^ This “dual lock‐and‐key” approach with DNA nanostructure offers enhancement over the delivery system via enhancing delivery specificity and preventing toxicity, so is of high importance for targeted siRNA delivery for cancer treatment.

**Figure 9:**
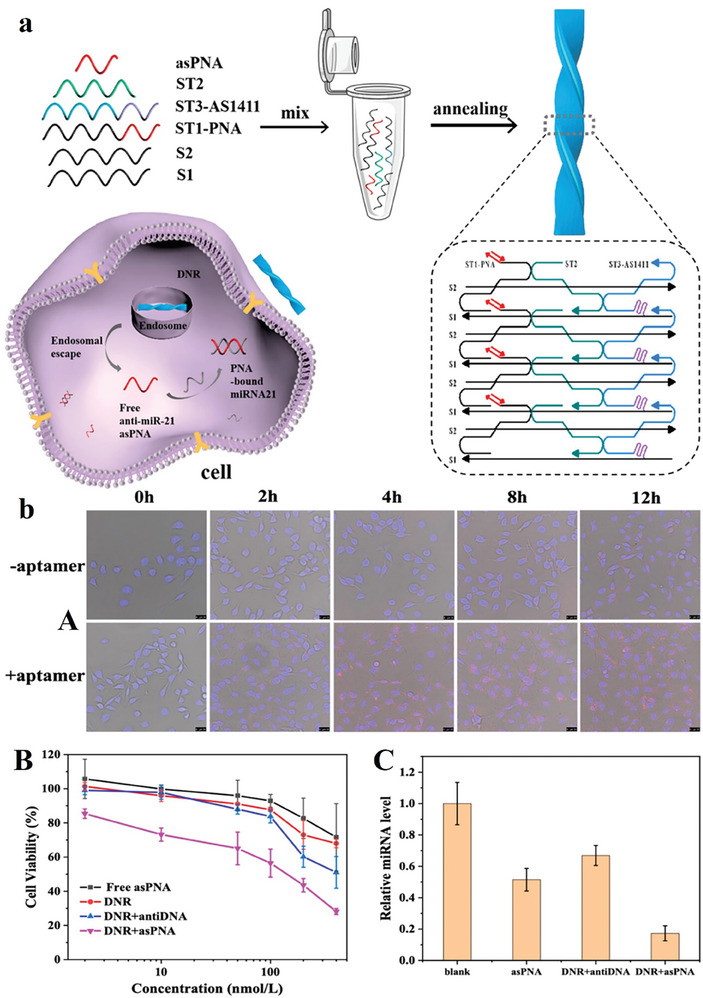
a) Assembly of DNA nanoribbon to cancer therapy with antisense. b) (A) KYSE150 esophageal carcinoma cells incubated with Cy5‐DNR+asPNA and Apt Cy5‐DNR+asPNA. The fluorescence shows DNR+asPNA comprising Cy5‐S1 (red), and cell nuclei have been stained by DAPI. The nanoplatform was internalized via the cells for 2 h. Besides, the DNR+asPNA structure with the aptamer increases the penetration into the cells. Scale bars are 25 µm. (B) The effects of various drugs on the growth of cells for 48 h. (C) RT‐qPCR examination of miR‐21 levels in KYSE150 cells with asPNA, DNR+antiDNA, and DNR+asPNA treatment during 2 days. Reproduced with permission from.^[^ ^103^ ^]^ Copyright 2023, Analytical Chemistry.

Targeted delivery of antisense peptide nucleic acids (asPNAs) to target tissues can overcome their control delivery challenges in biomedical applications.^[^ ^103^ ^]^ A study showed that an efficient DNA nanoribbon‐based drug delivery system can release the asPNA to prevent miRNA. AS1411 aptamer can connected to the nucleolin on the cell membrane for the detection of targeted cells and increasing the enrichment capacity of DNA nanoribbon. According to the biodegradability of DNA nanoribbons, the release of asPNA into the cytoplasm was done, and miR‐21 can target the programmed cell death 4 (PDCD4) gene, increase its expression, and enhance cell apoptosis. As shown in **Figure** **9**b Apt Cy5‐DNR+asPNA was competently internalized via the cells during 2 h and followed to accumulate in the cytoplasm at 12 h. The results showed the inflexible structure of DNR+asPNA with the aptamer increases the penetration via the membranes, causing effective cellular internalization. Besides, treatment of KYSE150 cells with DNA+asPNA prompted 70% cell apoptosis, while asPNA triggered less than a 25% apoptotic signal. This delivery system can used for antisense site‐specific therapy for cancer.^[^ ^103^ ^]^

### 2.4 DNA Nanoflower

**Figure 10:**
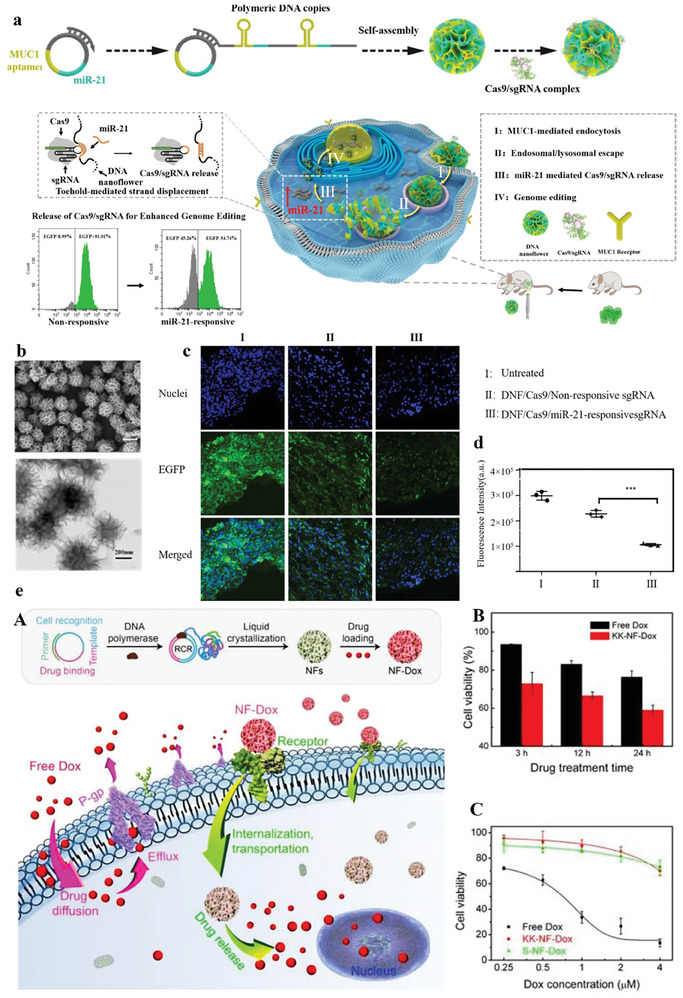
a) Schematic of microRNA‐responsive DNA nanoflower for delivery of Cas9/sgRNA and increased editing of genome. The CRISPR system was delivered from the nanostructure via a toehold‐mediated strand dislocation. In fact, the genome editing efficacy can be enhanced in the cells with greater miRNA expression levels. b) SEM (upper) and TEM (lower) imaging of the DNA nanoflower/Cas9/sgRNA nanoparticles. The nanostructure was uniformly sized with a size of 460 nm c) Fluorescent imaging of tumor segments. (A) Untreated; (B) DNF/Cas9/Non‐responsive sgRNA; (C) DNF/Cas9/miR‐21‐responsive sgRNA. d) the fluorescent intensity quantification in various groups ^***^ *p* < 0.001. Reproduced with permission from.^[^ ^105^ ^]^ Copyright 2020, Biomaterials. e) Multifunctional DNA nanoflowers as carriers to MDR cells for targeted drug delivery. (A) DOX was loaded into nanoflowers by encapsulation and DNA‐binding motifs. Nanoflowers facilitated the known, internalization, and drug delivery to MDR and chemosensitive cancer cells. (B) Cell viability of K562/MDR cells treated with free DOX and KK‐NF‐DOX, showing enhanced toxicity by using KK‐NF‐DOX. (C) KK‐NF‐DOX and S‐NF can prompt less toxicity in Ramos cells as a nontarget cell. Reproduced with permission from.^[^ ^106^ ^]^ Copyright 2015, Nano Research.

DNA nanoflower is a type of DNA hydrogel that does not depend on base‐pairing interactions. It is produced by PCR and compact packaging procedure. The nanostructure of this type has numerous benefits, such as tunable size, uncomplicated design and production, and resistance to enzymatic degradation. Researchers have constructed DNA nanoflowers integrated by the drug, and imaging agents.^[^ ^104^ ^]^ Researchers developed a rolling‐circle‐replication‐based assay to form DNA nanoflowers. The nanoflowers have shown numerous benefits as a cargo delivery carrier with great stability and efficient drug delivery. Functional DNA sequences could be integrated into the nanoflowers.^[^ ^104a^ ^]^ For example, Shi and co‐workers formed DNA nanoflowers comprising miR‐21 binding sequences. In fact, Cas9/sgRNA with a prolonged sequence that was 7 nucleotides shorter than miR‐21 was capable of being loaded on DNA nanoflowers among the stem‐loop of the sgRNA and the anti‐miR‐21 (**Figure** **10**a–d). When tumor cells were incubated with a miR‐21 caused by miR‐21 responsive Cas9/sgRNA delivery, the genome‐controlling efficacy was enhanced. In the cytoplasm, miR‐21 can exchange Cas9/sgRNA from DNA nanoflowers to deliver CRISPR‐Cas9, causing effective genome editing in Hela cells. In general, the design of a controlled delivery system for Cas9/sgRNA is key for enhancing genome editing efficacy. Besides, by integrating a stimulate‐responsive Cas9/sgRNA delivery system, more effective genome editing could be attained in the nanomaterials‐based CRISPR delivery system.^[^ ^105^ ^]^

One of the challenges in cancer therapy is multidrug resistance (MDR), which is triggered through drug efflux from tumor cells. Stimuli‐responsive drug delivery can overcome these challenges and nanoflower structures can be used for drug delivery to chemo‐sensitive and MDR cancer cells that avoid MDR in cancer, such as breast cancer cells. Nanoflowers with a size of 200 nm can be self‐assembled by magnesium pyrophosphate and DNA co‐precipitation produced by rolling circle replication, which Nanoflowers are integrated using aptamers to cancer cell recognition, and DOX‐binding DNA with the purpose of drug delivery. This structure has great drug‐loading capacity (71%), which is stable at pH 7.4, and drug delivery occurs under acidic or basic buffers (Figure 10e).^[^ ^106^ ^]^ Nanoflowers can release DOX into cancer cells, inhibiting drug efflux and increasing drug preservation in MDR cells. Besides, the developed structure induces toxicity in target MDR and chemo‐sensitive cells and decreases side effects.

**Figure 11:**
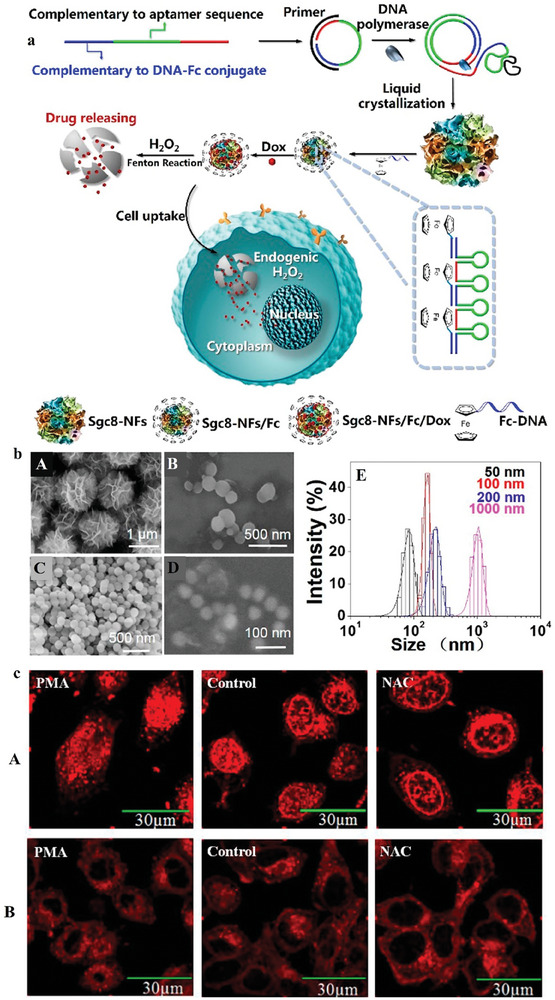
a) Schematic illustration of formation and self‐degradation by Fenton's reaction of Sgc8‐NFs‐Fc carrier. b) SEM images of developed systems with diverse sizes: (A) 1000 nm Sgc8‐NFs, (B) 200 nm, (C) 100 nm, (D) 50 nm Sgc8‐NFs‐Fc and (E) Different sizes of Sgc8‐NFs‐Fc. c) Distribution of DOX in cells treated with H_2_O_2_ stimulating PMA than MCF‐7 cells after incubation with (A) Sgc8‐NFs‐Fc/DOX and (B) Sgc8‐NFs3h/DOX. The treatment time of PMA was 60 min. Reproduced with permission from.^[^ ^107^ ^]^ Copyright 2019, American Chemical Society. PAM: Phorbol‐12‐myristate‐13‐ acetate; NAC: N‐acetylcysteine.

Nanoflowers also have high biocompatibility and biodegradability, which make them appropriate for delivery systems. Sgc8‐NFs‐F as a type of nanoflowers with a size of 1000 to 50 nm, can degrade to deliver DOX in the existence of H_2_O_2_. With aptamer integration permitted the Sgc8‐NFsFc complex to connect and internalize into tumor cells. The results established that this structure has efficient antitumor targeting efficacy providing a biodegradable delivery system for targeted drug delivery in cancers (**Figure** **11**a,b).^[^ ^107^ ^]^ As revealed in Figure 11c, MCF‐7 cells treated with PMA showed a high fluorescence signal in the nucleus and showed that the integration of Sgc8 in Sgc8‐ NFs‐Fc does not cooperate with its recognition capability and that Sgc8 can facilitate the accumulation of carriers in tumors. Nanoflowers, such as Sgc8‐NFs‐Fc can release its cargo to cells and decrease the common distribution of cargo in normal cells, though enhancing the therapeutic efficiency of DOX. Recently, RNA structures such as RNA and RNAi nanoflowers have become beautiful because of the variety of their functions. Through different assembly methods based on RNA nanotechnology, variations of therapeutic RNA structures protecting multiple therapeutic modules, such as ribozymes, aptamer, or siRNA, have been made. However, the quick progress of the RNA structures is delayed via main challenges, such as forming complex structures from molecular building blocks and targeting special ways for biomedical applications.^[^ ^108^ ^]^ Although, DNA nanoflowers are stable in physiological fluids according to the co‐precipitation of magnesium pyrophosphate and DNA products during the production procedure. However, this characteristic also reduces the bioavailability and efficiency of the cargo.^[^ ^107^ ^]^

### 2.5 DNA Cruciform

A DNA cruciform structure is a structure based on a DNA inverted repeat and considered via the existence of a four‐way junction wherein two of the hairpin structures are designed on each strand of the inverted repeat. The bases placed among the inverted repeats do not self‐pair.^[^ ^109^ ^]^ Up to now, various DNA nanostructures have been presented for the targeted delivery of drugs, such as DNA tetrahedron structure, dendrimer nanostructure, and DNA nanocentipede. However, the development of these structures is time‐consuming.^[^ ^110^ ^]^ Drugs such as DOX can connect to cruciform DNA nanostructure since it favorably intercalates among DNA base pairs. Complementary regions were formed which offered vast amounts of Cytosine and Guanine bases, causing to loading of the drug. It has been revealed that DOX could connect more resourcefully to dsDNA 5´‐ GC‐3´ or 5´‐CG‐3´.^[^ ^111^ ^]^ The loading of the drug into the cruciform structure was examined by evaluating the fluorescence strength of DOX after treatment with the nanostructure. A new cruciform structure was established for the targeted delivery of DOX to tumor cells. This DNA nanostructure was simple and could be formed rapidly. The DNA nanostructure was fabricated of two primers, such as FOXM1 and AS1411 aptamers. The existence of FOXM1 aptamer reduces the needed amount of drugs for effective tumor therapy. Therefore, the targeted drug delivery system has low toxicity for non‐target cells.^[^ ^53^ ^]^

### 2.6 DNA Nanocentipede and DNA Nanocockleburs

**Figure 12:**
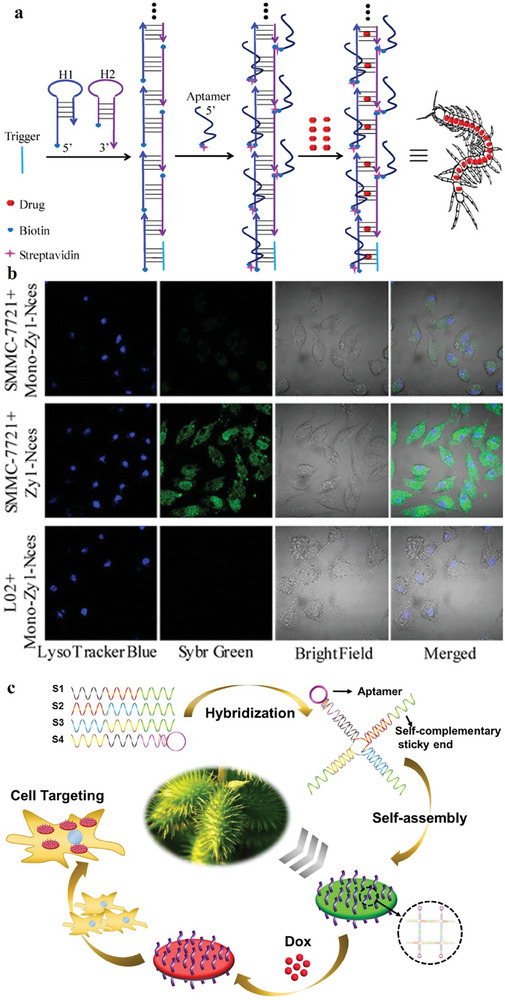
a) The self‐assembled aptamer‐based DNA nanocentipede as a drug nanocarrier. This structure comprises two DNA monomers (H1 and H2) in the trunk and the legs of nanocentipede as a function of targeting moieties to target cells. The trunk of the nanostructure was loaded with drugs. b) Comparison of the internalization of Zy1‐Nces and Mono‐Zy1‐Nces in different cells at 37 °C for 2 h. SMMC‐7721 cells showed high stronger green fluorescence signal than those treated with Mono‐Zy1‐Nces. (Scale bar: 20 µm). Reproduced with permission from.^[^ ^112^ ^]^ Copyright 2016, American Chemical Society. c) Schematic of the formation procedure of the aptamer‐nanocockleburs, in which four DNA strands are assembled into the sticky‐ended motif. At that time the sticky ends of each motif paired with other motifs’ sticky ends to assemble in aptamer‐nanocockleburs. Reproduced with permission from.^[54a]^ Copyright 2020, Colloids and Surfaces B: Biointerfaces.

DNA nanocentipede is deliberated as a potent drug delivery system. They have good geometrical flexibility. Their structure is similar to a centipede and comprises of “trunk” and “legs.” Its structural construction helps great loading capacity, as well as toxicity to target cancer cells especially.^[^ ^112^ ^]^ Researchers developed the role of the aptamers to target drug delivery as it delivers to the nanocentipede structure for its capability to show immunostimulatory CpG ODNs. To activate the nanocentipede with the purpose of delivery, the nanocentipede’ s “trunk” has been assembled using the HCR of two DNA monomers to which streptavidin‐conjugated ZY1 aptamers targeting SMMC‐7221 cells have been connected. The attachment of this nanostructure is increased by increasing the number of aptamer legs. In fact, the long trunk of the “DNA nanocentipede” was loaded with DOX.^[^ ^112^ ^]^ In addition, an enhancement in cellular internalization was recognized for multivalent nanocentipedes than monovalent (**Figure** **12**a,b). This platform showed the high capability to load DOX into the trunk of the nanocentipede, which is used as a significant platform for targeted drug delivery. On the other hand, Zy1‐Nces displayed great loading capacity and drug delivery. The results of the MTT assay exhibited improved cellular toxicity of the DOX‐loaded Zy1‐Nces to the targeted cells, but not normal cells.

Cockleburs can be delivered long distances from their parent plants to the pelt of animals due to their special surface structure, which is protected with hooked spines. The development of a cocklebur structure can be used as an aptamer‐modified DNA assembly for delivery of DOX to MCF‐7 cell lines. Figure 12c shows the construction method of aptamer‐nanocockleburs. Here, four complementary single DNA strands are sticky‐ended strands, assembled into a sticky‐ended motif. At that time, the sticky ends of every motif were paired with other sticky ends to assemble into aptamer‐nanocockleburs.^[^ ^54a^ ^]^ The aptamer‐nanostructure structures are prepared from DNA and can reduce the cost of other DNA structures.

## 3 Advantages, Challenges, and Future Perspective

DNA nanostructures present distinctive advantages across multiple dimensions. Initially, the configuration of nanomaterials has been recognized as a critical determinant of their biological functionality. The shapes and sizes of these structures play a pivotal role in governing their systemic circulation and clearance rates within the body. The inherent biodegradability and biocompatibility of DNA render DNA–nanostructures particularly intriguing as vehicles for drug delivery. Both dynamic and static DNA nanocarriers are meticulously engineered to exhibit passive or active release of payloads at specific anatomical sites.

**Figure 13:**
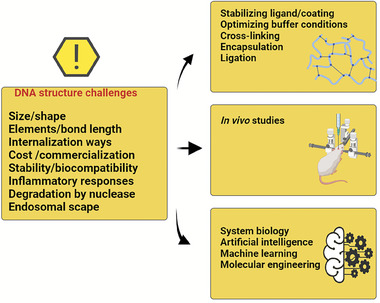
DNA structures, challenges, and solution ways for increased stability and future clinical applications. Created with BioRender.com.

Currently, most delivery systems consist of a combination of biomolecules with varying sizes, presenting challenges in accurately controlling the dimensions of these biomolecules. Inadequate research has been conducted regarding the impact of structure on the delivery system and generating diverse geometries from identical nanomaterials proves to be a formidable undertaking. Consequently, recent strides in DNA structure formation have yielded constructs that are monodisperse, characterized by controlled shapes and sizes. Noteworthy structures, such as tetrahedra, octahedra, and origami‐based objects, have revealed efficacy in transporting small‐molecule drugs, antibodies, CpG, aptamers, AuNPs, or siRNA for targeted delivery (**Figure** **13**).

Another salient feature of DNA nanostructures is their responsiveness to external stimuli, facilitating triggered drug delivery. Their programmability enables them to function as “logic gates,” responding predictably to stimuli. Furthermore, the integration of efficient molecules or ligands into these structures enhances their adaptability for specific purposes. These may include aptamers or antisense DNA, contributing to cell uptake with high specificity. Due to their unique targeting abilities, aptamers have found extensive utility as therapeutic agents.^[^ ^113^ ^]^ Different therapeutic agents can be encapsulated specifically with favorable payload in aptamer‐based DNA nanostructures for enhanced synergistic therapy (exceeding 30%) (**Table** **2**). This underscores the considerable potential of these targeted systems in biomedical applications.

**Table 2:** Summary of progress in aptamer‐based delivery strategies using DNA nanostructures.

DNA nanostructures	Cell/animal model	Aptamer	Toxicity of DNA nanostructure	Therapeutics agents	Loading capacity/efficiency	References
DNA origami	HeLa cells	M13/ sgc8 aptamer	No cytotoxicity	DOX	N/A	[115]
	Hela/ ADR cells	MUC‐ 1	Nontoxic even after incubation with cells at a high concentration (20 nM)	DOX	34.2%	[72]
	K299 cells	C2NP	N/A	DOX	N/A	[116]
	MCF‐7/ADR	MUC‐1	N/A	DOX	N/A	[76]
	4T1 cells/ 4T1 tumor‐bearing mice	AS1411	Nontoxic for normal cells	DOX	N/A	[75]
	MCF‐7 cells/ MDA‐MB‐231 cells	MUC‐1	No cytotoxicity	DOX	The loading capacities of Disc, Donut, and Sphere are 16.69% ± 2.20%, 21.76% ± 0.69%, and 21.97% ± 2.75%	[78]
	HeLa cell	Sgc8 aptamers	N/A	DOX	50%	[117]
DNA tetrahedron	MCF‐7	MUC‐1	No cytotoxicity	DOX	N/A	[118]
	HER2‐Positive Breast Cancer	Anit‐HApt aptamer	No‐cytotoxicity against the normal breast cell line (MCF10A cells)	Maytansine (DM1)	1.2%	[119]
	MCF‐7/ADR c	AS1411	No‐cytotoxicity at a high concentration of 300 nM for 24 h	DOX	N/A	[98]
	MCF‐7R	MUC1‐S4 (5′‐	N/A	DOX	40%	[120]
	MC38andCHOcell	PL1	No toxicity in vivo and in vitro	*Pcsk9* siRNA	N/A	[86]
	MCF‐7/MCF‐7 tumor‐bearing nude mice	MUC‐1	Low toxicity	DOX	55.99 ± 7.88	[96]
	HER2‐Positive Gastric Cancer	Anti HER2 aptamer (HAPt)	Low toxicity	Deruxtecan (Dxd)	3:1 drug/carrier ratio	[121]
	4T1 cell	AS1411	No cytotoxicity on 4T1 cells	DOX	N/A	[89]
DNA nanoribbon	KYSE150 cells	AS1411	N/A	Antisense peptide nucleic acid (asPNA)	N/A	[103]
DNA nanoflower	MCF7/MDR/MCF7/ K562 cells	N/A	Low toxicity	DOX	71.4%	[106]
	Hela cells	MUC1	N/A	Cas9/sgRNA	N/A	[105]
	MCF‐7 cells	sgc8	Low toxicity	DOX	0.131 micromole of DOX	[107]
DNA cruciform	A549 and 4T1 cells	AS1411 and FOXM1	N/A	DOX	N/A	[110]

Despite various advantages, these DNA nanostructures are associated with challenges in drug delivery systems, as follows: 1) One of the major limitations concerns the stability of DNA nanostructures in physiological situations. For example, decreases of Mg^2+^ in tissue culture medium could prompt denaturation of DNA origami structures. The great concentrations of the cations would care for the stability of DNA origami nanostructures in biological fluids. Researchers studied the stability of DNA origami nanostructures in low Mg^2+^ concentration. They establish that DNA origami stability relies on the accessibility of Mg^2+^ for selection electrostatic repulsion and the addition of cations, including sodium and sodium, was capable of stabilizing the DNA nanostructures.^[^ ^121^ ^]^

2) Another challenge is the degradation of DNA nanostructures by nuclease. Although studies showed the stability of DNA origami structures ssDNA or dsDNA, in a recent study, researchers developed the degradation kinetics of DNA box origami in serum. They established that the serum injured the structure of box origami and the digestion profile exhibited a quick collapse phase, that was monitored by a slow degradation phase.^[^ ^122^ ^]^ One of the ways to increase nuclease resistance under serum conditions is polymer‐based nanomaterials have been applied to coat DNA nanostructures. Researchers considered the susceptibility of DNA‐helix to degradation via different nucleases, as well as detecting digestion for T7 endonuclease and DNase I as the most plentiful nuclease in serum and blood. Although, DNase I prompted degradation was slower for the DNA origami compared to the plasmid DNA.^[^ ^123^ ^]^ One of the solutions is the DNA nanostructures’ design with high stability by making them very rigid for destroying nuclease binding. The activity of a DNA nanostructure depends on its uptake and stability, besides drug loading efficacy and release kinetics, which have been affected by similar design factors. Thus, other properties should be considered, such as effective drug intercalation over serum stability. Kim and co‐workers used an orthogonal base‐pairing system, that is, l‐DNA instead of d‐DNA, for their DNA nanostructure vehicle to evade unwanted interaction among the carrier and the aptamer cargo.^[^ ^124^ ^]^ This modification is controlled to intensely increase intracellular delivery rates.

3) There are different variables on which the cellular uptake efficacy of DNA nanostructures depends on shapes and size, type of cell, and distribution ways. DNA nanostructures can enter cells in five ways, such as clathrin‐ and caveolin‐mediated endocytosis, phagocytosis, clathrin‐ and caveolin‐independent and macropinocytosis. However, which shape, and size of DNA nanostructures offer which ways are still not clear. Moreover, we need some molecules or ways to confirm the specificity of the different pathways. With the use of machine learning techniques, artificial intelligence or structure‐activity‐application regression analytical models with inputs (e.g., geometry, synthesis) and outcomes (e.g., drug release) will allow researchers to select the specific DNA origami for biomedical applications.^[^ ^125^ ^]^

4) Although DNA nanostructures are intrinsically biocompatible and perform as main players in different procedures, they can produce inflammatory responses. Researchers detected immune activation in mice splenocytes treated with DNA origami by observing their cytokine fabrication, such as interleukin 6 (IL‐6) and interleukin12 (IL‐12) levels.^[^ ^126^ ^]^ In recent times, a study showed that DNA origami nanostructures stimulated immunogenicity and the immune response reduced over time.^[^ ^127^ ^]^ Chemical modification of the scaffold and main DNA strands could be a hopeful key to decreasing the immunogenicity of DNA origami.

5) Clinical applications and commercialization are other important challenges that should be investigated. One of the critical issues is the long‐term storage and long‐term durability of these DNA nanostructures. Lyophilization helps the long‐term storage of DNA nanostructures. Also, numerous nucleic acid‐based drugs containing synthetic oligonucleotides have been approved by the FDA and are currently in clinical laboratories, we expect that fully synthetic DNA nanostructures will be more effective based on genomic scaffolds and have fewer obstacles to overwhelmed on their way to FDA approval. Although, for therapeutic DNA nanostructures to clinical trials, important savings will be needed according to the large costs of small‐scale GMP‐obedient oligonucleotide synthesis. Although with approval of DNA nanostructures arrives on the market, we anticipate manufacturing costs to reasonable levels, therefore interpretation of DNA nanostructure‐based delivery system hopeful therapeutics for the treatment of different diseases and cancers.

6) The synthesis of DNA nanostructures poses a notable challenge owing to its elevated cost, hindering scalability in preparation. Furthermore, the utilization of aptamers encounters obstacles, notably excessive clearance, particularly for unmodified aptamers. Persistent concerns regarding production costs necessitate the capability to prepare functional DNA nanostructures on a larger scale while maintaining high purity. Despite the existence of cost‐effective purification methods demonstrated at the laboratory level, there is a dearth of evidence regarding their scalability. Various strategies, including agarose‐gel‐based separation and ultracentrifugation, have been explored to tackle these challenges. In addressing the challenge of reducing kidney filtration, a potential solution involves conjugating aptamers with nanoparticles. The generation of aptamers, a process achieved through SELEX methods, is time‐consuming, and influenced by factors such as alterations in PCR methods and library determination. Although newer methods like Capillary Electrophoresis‐SELEX (CE‐SELEX) hold promise in shortening experimental periods, their commercialization remains pending. To mitigate the cost of DNA chemical synthesis on a larger scale, alternative methods such as in‐cell production, fermentation, RCA, and asymmetric PCR have been explored. High‐cell‐density bioreactors via fermentation have proven effective in generating substantial DNA quantities for nanostructure production. Modifications to PCR protocols and enzymatic digestion of RCA products have enabled the preparation of both long single‐stranded and short oligonucleotides. Despite progress, a significant gap exists before DNA‐nanostructure drug vehicles can competitively match the cost efficiency of polymer materials, which can be less than $1 per gram.^[^ ^2a^ ^]^ Ongoing efforts to optimize these alternative methods offer potential avenues for reducing costs and enhancing the feasibility of DNA‐based nanostructure applications in practical biomedical settings.

7) The issue of biosafety assumes significance when considering the engineered transformation of DNA into nanostructures, despite its inherent biodegradability and biocompatibility. While research endeavors in this realm persist, several fundamental inquiries remain unanswered. Specifically, there is a need to elucidate the ultimate fate of metal or carbon elements within metal‐assisted DNA nanostructures or nanoparticle‐templated. Questions concerning the potential toxicity of these metal elements to cellular entities and the sustainability of the resulting nanomaterials are central to this discourse. In metal‐assisted DNA nanostructures or nanoparticle‐templated, what can be the final fate of metal or carbon elements, and are these elements potentially toxic to cells? Do concerns exist regarding the sustainability of nanomaterials formed through the engineering of DNA into nanostructures? To what degree do life cycle assessments play a role in assessing the human body and environmental impacts within the context of drug delivery?

Therefore, we need more research to focus on explaining and controlling the synthesis and molecular mechanisms of the stability of DNA nanostructures under physiological conditions. Several strategies that can be used for enhanced controlling DNA nanostructures stability and immunogenicity have been developed, such as chemical and photo‐crosslinking modifications, enzymatic ligation, protein encapsulation, and surface coatings. For example, the toxicity of polymer‐origami complexes shows low toxicity in different concentration levels. Besides, with catting of brick‐like DNA origamis with the polymers, researchers can regulate the number of cationic polymers and control the enzyme kinetics of the complexes.^[^ ^128^ ^]^

Although many of these assays have resulted in major enhancements in the stability of DNA nanostructures and cellular uptake, they have a considerable risk of interfering with the therapeutic activity, loading, and release of cargo. For example, with coating, the connecting attraction of surface‐bound aptamers can be decreased by the use of these coatings. Moreover, most of the coatings are not compatible with the conversion of DNA nanostructures. In conclusion, coating DNA nanostructures will limit entrance to encapsulated cargo and thus affect drug‐loading and release properties. In fact, modifying DNA nanostructure's stability, immunogenicity, drug loading and delivery, and analyte binding denotes the biggest challenge that biomedical DNA nanotechnology presently faces.

## 4 Conclusion

In recent years, researchers have developed innovative assays to assess the stability of DNA nanostructures in serum, thereby enhancing their cellular uptake. This advancement is particularly noteworthy, given that DNA nanostructures demonstrate significant potential in applications such as imaging and drug delivery systems, owing to their inherent robustness. The efficacy of these structures is underscored by their compatibility with a diverse range of probes, ligands, and drug types. Notably, certain structures have exhibited efficient instantiation in vivo. The integration of DNA nanostructures with aptamers presents precise tools for the regulation of targeted delivery systems. While the field of drug delivery within DNA nanostructures is relatively nascent, deriving insights from additional research endeavors within the broader domain of drug delivery is imperative. Rather than exclusively concentrating on this emerging field, heightened attention should be directed toward comprehending disease biology and advancing personalized medicine through the application of DNA nanotechnology. Consequently, comprehensive studies conducted under in vivo conditions and across various animal models are warranted, with the replication of experiments in multiple laboratories.

As mentioned earlier, DNA nanostructures, employed as an entirely biologically derived and sustainable approach to drug delivery, warrant attention due to their incorporation of carbon and metal nanomaterials, strategically aimed at enhancing efficacy. Despite the recognition of these nanoparticles as biocompatible, a fundamental query arises regarding their potential accumulation within the human body. Additionally, a thorough examination is warranted concerning the sustainability of the synthesis methods applied to these nanomaterials. The accumulation of carbon dioxide and carbon in the environment, serving as a direct or indirect catalyst for numerous diseases, including cancer, underscores the need for careful consideration. Therefore, prudent attention must be given to the synthesis methods of carbon and metal nanomaterials, as well as their potential environmental release. Oversight in these aspects may inadvertently exacerbate existing diseases, rendering futile the concerted efforts invested in enhancing drug delivery mechanisms. Anticipating an impending paradigm shift, the focus extends beyond refining drug delivery within DNA nanostructure systems to ensuring the sustainability of applied synthesis methods. This paradigm entails the critical evaluation of nano auxiliary materials and the optimization of energy consumption, coupled with comprehensive life cycle assessments in both biological and environmental contexts. Such a strategic realignment seamlessly aligns with the imperative of adhering to a zero‐carbon net policy.

## Conflict of Interest

The authors declare no conflict of interest.

## Author Contributions

The manuscript was written through the contributions of all authors. All authors have approved the final version of the manuscript.

## References

[1] a) E. Jergens , J. O. Winter , Curr. Opin. Biotechnol. 2022, 74, 278; 35026622 10.1016/j.copbio.2021.12.010

[2] a) Q. Hu , H. Li , L. Wang , H. Gu , C. Fan , Chem. Rev. 2019, 119, 6459; 29465222 10.1021/acs.chemrev.7b00663

[3] S. Jiang , Z. Ge , S. Mou , H. Yan , C. Fan , Chem 2021, 7, 1156.

[4] a) K. Matange , J. M. Tuck , A. J. Keung , Nat. Commun. 2021, 12, 1358; 33649304 10.1038/s41467-021-21587-5 PMC7921107

[5] a) W. Ma , Y. Zhan , Y. Zhang , C. Mao , X. Xie , Y. Lin , Signal Transduct. Targeted Therapy 2021, 6, 351; 10.1038/s41392-021-00727-9 PMC8497566 34620843

[6] a) P. Zhan , A. Peil , Q. Jiang , D. Wang , S. Mousavi , Q. Xiong , Q. Shen , Y. Shang , B. Ding , C. Lin , Y. Ke , N. Liu , Chem. Rev. 2023, 123, 3976; 36990451 10.1021/acs.chemrev.3c00028 PMC10103138

[7] a) A. Rajwar , S. R. Shetty , P. Vaswani , V. Morya , A. Barai , S. Sen , M. Sonawane , D. Bhatia , ACS Nano 2022, 16, 10496; 35715010 10.1021/acsnano.2c01382

[8] F. Wang , B. Willner , I. Willner , Curr. Opin. Biotechnol. 2013, 24, 562. 23477850 10.1016/j.copbio.2013.02.005

[9] D. Fu , J. Reif , 2021, 11, 2624.

[10] K. E. Bujold , A. Lacroix , H. F. Sleiman , Chem 2018, 4, 495.

[11] a) R. E. Farrell , in RNA Methodologies (Ed: R. E. Farrell ), Academic Press, San Diego 2010;

[12] X. Fu , H. Yang , Z. Li , N.‐C. Liu , P.‐S. Lee , K. Li , S. Li , M. Ding , J. S. Ho , Y.‐C. E. Li , ACS Biomater. Sci. Eng. 2021, 9, 2129. 34297522 10.1021/acsbiomaterials.1c00591

[13] a) J. Hahn , S. F. J. Wickham , W. M. Shih , S. D. Perrault , ACS Nano 2014, 8, 8765; 25136758 10.1021/nn503513p PMC4174095

[14] a) C. Kielar , Y. Xin , B. Shen , M. A. Kostiainen , G. Grundmeier , V. Linko , A. Keller , Angew. Chem. 2018, 130, 9614; 10.1002/anie.201802890 29799663

[15] L. Cordsmeier , M. B. Hahn , ChemBioChem 2022, 23, 202200391. 10.1002/cbic.202200391 PMC9826032 35972228

[16] K. Matange , J. M. Tuck , A. J. Keung , Nat. Commun. 2021, 12, 1358. 33649304 10.1038/s41467-021-21587-5 PMC7921107

[17] N. D. F. Grindley , K. L. Whiteson , P. A. Rice , Annu. Rev. Biochem. 2006, 75, 567. 16756503 10.1146/annurev.biochem.73.011303.073908

[18] a) M. J. Mitchell , M. M. Billingsley , R. M. Haley , M. E. Wechsler , N. A. Peppas , R. Langer , Nat. Rev. Drug Discovery 2021, 20, 101; 33277608 10.1038/s41573-020-0090-8 PMC7717100

[19] a) K. Samejima , W. C. Earnshaw , Nat. Rev. Mol. Cell Biol. 2005, 6, 677; 16103871 10.1038/nrm1715

[20] A. R. Chandrasekaran , Nat. Reviews Chem. 2021, 5, 225. 10.1038/s41570-021-00251-y PMC7873672 33585701

[21] J. Hahn , S. F. Wickham , W. M. Shih , S. D. Perrault , ACS Nano 2014, 8, 8765. 25136758 10.1021/nn503513p PMC4174095

[22] S. Goltry , N. Hallstrom , T. Clark , W. Kuang , J. Lee , C. Jorcyk , W. B. Knowlton , B. Yurke , W. L. Hughes , E. Graugnard , Nanoscale 2015, 7, 10382. 25959862 10.1039/c5nr02283e PMC4457601

[23] E. Benson , A. Mohammed , J. Gardell , S. Masich , E. Czeizler , P. Orponen , B. Högberg , Nature 2015, 523, 441. 26201596 10.1038/nature14586

[24] A. Cherepanova , S. Tamkovich , D. Pyshnyi , M. Kharkova , V. Vlassov , P. Laktionov , J. Immunol. Methods 2007, 325, 96. 17618645 10.1016/j.jim.2007.06.004

[25] C. E. Castro , F. Kilchherr , D.‐N. Kim , E. L. Shiao , T. Wauer , P. Wortmann , M. Bathe , H. Dietz , Nat. Methods 2011, 8, 221. 21358626 10.1038/nmeth.1570

[26] E.‐C. Wamhoff , A. Romanov , H. Huang , B. J. Read , E. Ginsburg , G. A. Knappe , H. M. Kim , N. P. Farrell , D. J. Irvine , M. Bathe , ACS Nano 2022, 16, 8954. 35640255 10.1021/acsnano.1c11575 PMC9649841

[27] S.‐T. Wang , M. A. Gray , S. Xuan , Y. Lin , J. Byrnes , A. I. Nguyen , N. Todorova , M. M. Stevens , C. R. Bertozzi , R. N. Zuckermann , Proc. Natl. Acad. Sci. U.S.A. 2020, 117, 6339. 32165539 10.1073/pnas.1919749117 PMC7104344

[28] S. D. Perrault , W. M. Shih , ACS Nano 2014, 8, 5132. 24694301 10.1021/nn5011914 PMC4046785

[29] F. M. Anastassacos , Z. Zhao , Y. Zeng , W. M. Shih , J. Am. Chem. Soc. 2020, 142, 3311. 32011869 10.1021/jacs.9b11698

[30] H. B. D. Thai , K.‐R. Kim , K. T. Hong , T. Voitsitskyi , J.‐S. Lee , C. Mao , D.‐R. Ahn , ACS Cent. Sci. 2020, 6, 2250. 33376785 10.1021/acscentsci.0c00763 PMC7760472

[31] V. Cassinelli , B. Oberleitner , J. Sobotta , P. Nickels , G. Grossi , S. Kempter , T. Frischmuth , T. Liedl , A. Manetto , Angew. Chem., Int. Ed. 2015, 54, 7795. 10.1002/anie.201500561 25980669

[32] T. Gerling , M. Kube , B. Kick , H. Dietz , Sci. Adv. 2018, 4, eaau1157. 30128357 10.1126/sciadv.aau1157 PMC6097813

[33] Y. Ahmadi , E. De Llano , I. Barišić , Nanoscale 2018, 10, 7494. 29637957 10.1039/c7nr09461b

[34] E.‐C. Wamhoff , H. Huang , B. J. Read , E. Ginsburg , W. R. Schief , N. Farrell , D. J. Irvine , M. Bathe , bioRxiv 2020, 2020.

[35] C. Guan , X. Zhu , C. Feng , Biomolecules 2021, 11, 1855. 34944499 10.3390/biom11121855 PMC8699395

[36] I. Khalil , A. Hashem , A. R. Nath , N. Muhd Julkapli , W. A. Yehye , W. J. Basirun , Mol. Cell. Probes 2021, 59, 101758. 34252563 10.1016/j.mcp.2021.101758

[37] P. Charoenphol , H. Bermudez , Mol. Pharmaceutics 2014, 11, 1721. 10.1021/mp500047b PMC4018137 24739136

[38] a) G.‐M. Han , B. Liu , D.‐M. Kong , L.‐N. Zhu , Mater. Chem. Front. 2023, 7, 6345;

[39] J. Ji , D. Karna , H. Mao , Chem. Soc. Rev. 2021, 50, 11966. 34499072 10.1039/d1cs00250c PMC8966688

[40] J. Bush , S. Singh , M. Vargas , E. Oktay , C.‐H. Hu , R. Veneziano , Molecules 2020, 25, 3386. 32722650 10.3390/molecules25153386 PMC7435391

[41] C. Li , Y. Wang , P.‐F. Li , Q. Fu , Acta Biomater. 2023, 160, 1. 36764595 10.1016/j.actbio.2023.02.005

[42] M. Xu , C. Zhang , C. Zhang , Y. Zhao , Z. Qi , C. Fan , J. Chao , B. Wei , ACS Mater. Lett. 2020, 2, 1322.

[43] Y. Xu , Z. Lv , C. Yao , D. Yang , Biomater. Sci. 2022, 10, 3054. 35535967 10.1039/d2bm00445c

[44] S. Sun , Y. Yang , D. Li , J. Zhu , J. Am. Chem. Soc. 2019, 141, 19524. 31789023 10.1021/jacs.9b08737

[45] S. Xing , D. Jiang , F. Li , J. Li , Q. Li , Q. Huang , L. Guo , J. Xia , J. Shi , C. Fan , ACS Appl. Mater. Interfaces 2015, 7, 13174. 25345465 10.1021/am505592e

[46] a) Y. Ryu , C. Am Hong , Y. Song , J. Beak , B. Am Seo , J.‐j. Lee , H.‐S. Kim , Nanoscale 2020, 12, 4975; 32057052 10.1039/c9nr08519j

[47] T. Yata , Y. Takahashi , M. Tan , K. Hidaka , H. Sugiyama , M. Endo , Y. Takakura , M. Nishikawa , Sci. Rep. 2015, 5, 14979. 26462616 10.1038/srep14979 PMC4604513

[48] D. Fu , J. Reif , Appl. Sci. 2021, 11, 2624.

[49] Y. Zhang , X. Tian , Z. Wang , H. Wang , F. Liu , Q. Long , S. Jiang , Front. Mol. Biosci. 2023, 10. 10.3389/fmolb.2023.1239952 PMC10440542 37609372

[50] F. Liu , X. Liu , W. Gao , L. Zhao , Q. Huang , T. Arai , Iscience 2023, 26. 10.1016/j.isci.2023.106208 PMC9982283 36876133

[51] a) W. Wang , M. Lin , W. Wang , Z. Shen , Z.‐S. Wu , Bioactive Materials 2024, 33, 279; 38076646 10.1016/j.bioactmat.2023.10.025 PMC10701289

[52] P. Fu , H. Chen , L. Ouyang , L. Li , Y. Wang , S. Qian , Z. Cao , K. Wu , J. Chao , J. Zheng , Anal. Chem. 2022, 95, 1811. 10.1021/acs.analchem.2c04760 36542541

[53] K. Abnous , N. M. Danesh , M. Ramezani , F. Charbgoo , A. Bahreyni , S. M. Taghdisi , Expert Opinion Drug Del. 2018, 15, 1045. 10.1080/17425247.2018.1530656 30269603

[54] a) S. Sun , N. Cheraga , H.‐N. Jiang , Q.‐R. Xiao , P.‐C. Gao , Y. Wang , Y.‐Y. Wei , X.‐W. Wang , Y. Jiang , Colloids Surf., B 2020, 186, 110733; 10.1016/j.colsurfb.2019.110733 31864113

[55] a) A. Lacroix , T. G. W. Edwardson , M. A. Hancock , M. D. Dore , H. F. Sleiman , J. Am. Chem. Soc. 2017, 139, 7355; 28475327 10.1021/jacs.7b02917

[56] J. Liu , T. Wei , J. Zhao , Y. Huang , H. Deng , A. Kumar , C. Wang , Z. Liang , X. Ma , X.‐J. Liang , Biomaterials 2016, 91, 44. 26994877 10.1016/j.biomaterials.2016.03.013

[57] A. Jabbari , E. Sameiyan , E. Yaghoobi , M. Ramezani , M. Alibolandi , K. Abnous , S. M. Taghdisi , Int. J. Pharm. 2023, 646, 123448. 37757957 10.1016/j.ijpharm.2023.123448

[58] X. Dai , H. Cheng , Z. Bai , J. Li , J. Cancer 2017, 8, 3131. 29158785 10.7150/jca.18457 PMC5665029

[59] a) S. Xie , W. Sun , T. Fu , X. Liu , P. Chen , L. Qiu , F. Qu , W. Tan , J. Am. Chem. Soc. 2023, 145, 7677; 36987838 10.1021/jacs.3c00841

[60] a) G. Vindigni , S. Raniolo , F. Iacovelli , V. Unida , C. Stolfi , A. Desideri , S. Biocca , Pharmaceutics 2021, 13, 1671; 34683964 10.3390/pharmaceutics13101671 PMC8541364

[61] M. Chang , C.‐S. Yang , D.‐M. Huang , ACS Nano 2011, 5, 6156. 21732610 10.1021/nn200693a

[62] Z. Xia , P. Wang , X. Liu , T. Liu , Y. Yan , J. Yan , J. Zhong , G. Sun , D. He , Biochemistry 2016, 55, 1326. 26789283 10.1021/acs.biochem.5b01181

[63] E. Yaghoobi , T. Zavvar , M. Ramezani , M. Alibolandi , S. Rahimzadeh Oskuei , M. Zahiri , M. Alinezhad Nameghi , K. Abnous , S. M. Taghdisi , J. Drug Targeting 2022, 30, 1106. 10.1080/1061186X.2022.2094387 35736221

[64] L. Sala , T. Perecko , O. Mestek , D. Pinkas , T. Homola , J. Kočišek , ACS Appl. Nano Mater. 2022, 5, 13267.

[65] Q. Zhang , Q. Jiang , N. Li , L. Dai , Q. Liu , L. Song , J. Wang , Y. Li , J. Tian , B. Ding , Y. Du , ACS Nano 2014, 8, 6633. 24963790 10.1021/nn502058j

[66] E. S. Andersen , M. Dong , M. M. Nielsen , K. Jahn , R. Subramani , W. Mamdouh , M. M. Golas , B. Sander , H. Stark , C. L. P. Oliveira , J. S. Pedersen , V. Birkedal , F. Besenbacher , K. V. Gothelf , J. Kjems , Nature 2009, 459, 73. 19424153 10.1038/nature07971

[67] C. Ouyang , S. Zhang , C. Xue , X. Yu , H. Xu , Z. Wang , Y. Lu , Z.‐S. Wu , J. Am. Chem. Soc. 2020, 142, 1265. 31895985 10.1021/jacs.9b09782

[68] S. Zhao , R. Tian , J. Wu , S. Liu , Y. Wang , M. Wen , Y. Shang , Q. Liu , Y. Li , Y. Guo , Z. Wang , T. Wang , Y. Zhao , H. Zhao , H. Cao , Y. Su , J. Sun , Q. Jiang , B. Ding , Nat. Commun. 2021, 12, 358. 33441565 10.1038/s41467-020-20638-7 PMC7807036

[69] N. Joseph , A. Shapiro , E. Gillis , S. Barkey , A. Abu‐Horowitz , I. Bachelet , B. Mizrahi , Sci. Rep. 2023, 13, 19567. 37949918 10.1038/s41598-023-46351-1 PMC10638432

[70] J. Spratt , J. M. Dias , C. Kolonelou , G. Kiriako , E. Engström , E. Petrova , C. Karampelias , I. Cervenka , N. Papanicolaou , A. Lentini , B. Reinius , O. Andersson , E. Ambrosetti , J. L. Ruas , A. I. Teixeira , Nat. Nanotechnol. 2023, 19, 237. 37813939 10.1038/s41565-023-01507-y PMC10873203

[71] S. Li , Q. Jiang , S. Liu , Y. Zhang , Y. Tian , C. Song , J. Wang , Y. Zou , G. J. Anderson , J.‐Y. Han , Y. Chang , Y. Liu , C. Zhang , L. Chen , G. Zhou , G. Nie , H. Yan , B. Ding , Y. Zhao , Nat. Biotechnol. 2018, 36, 258. 29431737 10.1038/nbt.4071

[72] Q. Pan , C. Nie , Y. Hu , J. Yi , C. Liu , J. Zhang , M. He , M. He , T. Chen , X. Chu , ACS Appl. Mater. Interfaces 2020, 12, 400. 31815420 10.1021/acsami.9b20707

[73] a) P. Wang , M. A. Rahman , Z. Zhao , K. Weiss , C. Zhang , Z. Chen , S. J. Hurwitz , Z. G. Chen , D. M. Shin , Y. Ke , J. Am. Chem. Soc. 2018, 140, 2478; 29406750 10.1021/jacs.7b09024 PMC7261494

[74] S. M. Taghdisi , N. M. Danesh , M. Ramezani , R. Yazdian‐Robati , K. Abnous , Mol. Pharmaceutics 2018, 15, 1972. 10.1021/acs.molpharmaceut.8b00124 29669200

[75] M. Li , G. Yang , Y. Zheng , J. Lv , W. Zhou , H. Zhang , F. You , C. Wu , H. Yang , Y. Liu , J. Nanobiotechnol. 2023, 21, 186. 10.1186/s12951-023-01953-9 PMC10257293 37301952

[76] L. Song , Q. Jiang , J. Liu , N. Li , Q. Liu , L. Dai , Y. Gao , W. Liu , D. Liu , B. Ding , Nanoscale 2017, 9, 7750. 28581004 10.1039/c7nr02222k

[77] a) J. Liu , L. Song , S. Liu , S. Zhao , Q. Jiang , B. Ding , Angew. Chem. 2018, 57, 15486; 30288887 10.1002/anie.201809452

[78] A. Udomprasert , C. Wootthichairangsan , R. Duangrat , S. Chaithongyot , Y. Zhang , R. Nixon , W. Liu , R. Wang , M. Ponglikitmongkol , T. Kangsamaksin , ACS Appl. Bio Mater. 2022, 5, 2262. 10.1021/acsabm.2c00114 35500214

[79] J. Zhang , Y.‐X. Cui , X.‐N. Feng , M. Cheng , A.‐N. Tang , D.‐M. Kong , ACS Appl. Mater. Interfaces 2019, 11, 39624. 31573175 10.1021/acsami.9b14186

[80] S. Chaithongyot , R. Duangrat , C. Wootthichairangsan , R. Hanchaina , A. Udomprasert , T. Kangsamaksin , Mater. Lett. 2020, 260, 126952.

[81] J. Yan , X. Zhan , Z. Zhang , K. Chen , M. Wang , Y. Sun , B. He , Y. Liang , J. Nanobiotechnol. 2021, 19, 412. 10.1186/s12951-021-01164-0 PMC8650297 34876145

[82] C. Guan , X. Zhu , C. Feng , Biomolecules 2021, 1855. 34944499 10.3390/biom11121855 PMC8699395

[83] a) Y. Zhan , W. Ma , Y. Zhang , C. Mao , X. Shao , X. Xie , F. Wang , X. Liu , Q. Li , Y. Lin , ACS Appl. Mater. Interfaces 2019, 11, 15354; 30924334 10.1021/acsami.9b03449

[84] H. Han , Methods Mol. Biol. 2018, 1706, 293. 29423805 10.1007/978-1-4939-7471-9_16 PMC6743327

[85] D. Xiao , Y. Li , T. Tian , T. Zhang , S. Shi , B. Lu , Y. Gao , X. Qin , M. Zhang , W. Wei , Y. Lin , ACS Appl. Mater. Interfaces 2021, 13, 6109. 33497198 10.1021/acsami.0c23005

[86] W. Guo , H. Gao , H. Li , S. Ge , F. Zhang , L. Wang , H. Shi , A. Han , ACS Appl. Mater. Interfaces 2022, 14, 31634. 35817627 10.1021/acsami.2c06001 PMC9305706

[87] T. Zhang , T. Tian , R. Zhou , S. Li , W. Ma , Y. Zhang , N. Liu , S. Shi , Q. Li , X. Xie , Y. Ge , M. Liu , Q. Zhang , S. Lin , X. Cai , Y. Lin , Nat. Protoc. 2020, 15, 2728. 32669637 10.1038/s41596-020-0355-z

[88] a) K. R. Kim , D. R. Kim , T. Lee , J. Y. Yhee , B. S. Kim , I. C. Kwon , D. R. Ahn , Chem. Commun. 2013, 49, 2010; 10.1039/c3cc38693g 23380739

[89] Q. Wang , Y. Ma , Z. Lu , H. Yu , Z. Li , ACS Appl. Nano Mater. 2022, 5, 101.

[90] X. Liu , H. Yan , Y. Liu , Y. Chang , Small 2011, 7, 1673. 21538862 10.1002/smll.201002292

[91] H. Wei , F. Li , T. Xue , H. Wang , E. Ju , M. Li , Y. Tao , Bioact. Mater. 2023, 28, 50. 37214257 10.1016/j.bioactmat.2023.04.024 PMC10199164

[92] T. Wu , J. Liu , M. Liu , S. Liu , S. Zhao , R. Tian , D. Wei , Y. Liu , Y. Zhao , H. Xiao , B. Ding , Angew. Chem., Int. Ed. 2019, 58, 14224. 10.1002/anie.201909345 31389144

[93] a) K. Paunovska , D. Loughrey , J. E. Dahlman , Nat. Rev. Genet. 2022, 23, 265; 34983972 10.1038/s41576-021-00439-4 PMC8724758

[94] a) D. Huo , X. Jiang , Y. Hu , Adv. Mater. 2020, 32, 1904337; 10.1002/adma.201904337 31663198

[95] W. Fu , C. You , L. Ma , H. Li , Y. Ju , X. Guo , S. Shi , T. Zhang , R. Zhou , Y. Lin , ACS Appl. Mater. Interfaces 2019, 11, 39525. 31601097 10.1021/acsami.9b13829

[96] X. Han , Y. Jiang , S. Li , Y. Zhang , X. Ma , Z. Wu , Z. Wu , X. Qi , Nanoscale 2019, 11, 339. 10.1039/c8nr05546g 30534748

[97] L. Meng , W. Ma , M. Zhang , R. Zhou , Q. Li , Y. Sun , W. Fu , X. Cai , Y. Lin , Appl. Mater. Today 2021, 23, 101010.

[98] X. Liu , L. Wu , L. Wang , W. Jiang , Talanta 2018, 179, 356. 29310244 10.1016/j.talanta.2017.11.034

[99] L. L. Li , W. Y. Lv , Y. T. Xu , Y. F. Li , C. M. Li , C. Z. Huang , Anal. Chem. 2022, 94, 4399. 35230818 10.1021/acs.analchem.1c05327

[100] a) G. Chen , D. Liu , C. He , T. R. Gannett , W. Lin , Y. Weizmann , J. Am. Chem. Soc. 2015, 137, 3844; 25622178 10.1021/ja512665z

[101] Z. Zhang , M. M. Ali , M. A. Eckert , D.‐K. Kang , Y. Y. Chen , L. S. Sender , D. A. Fruman , W. Zhao , Biomaterials 2013, 34, 9728. 24044994 10.1016/j.biomaterials.2013.08.079

[102] K. Ren , Y. Liu , J. Wu , Y. Zhang , J. Zhu , M. Yang , H. Ju , Nat. Commun. 2016, 7, 13580. 27882923 10.1038/ncomms13580 PMC5476801

[103] P. Fu , H. Chen , L. Ouyang , L. Li , Y. Wang , S. Qian , Z. Cao , K. Wu , J. Chao , J. Zheng , Anal. Chem. 2023, 95, 1811. 10.1021/acs.analchem.2c04760 36542541

[104] a) Y. Lv , R. Hu , G. Zhu , X. Zhang , L. Mei , Q. Liu , L. Qiu , C. Wu , W. Tan , Nat. Protoc. 2015, 10, 1508; 26357007 10.1038/nprot.2015.078 PMC4927333

[105] J. Shi , X. Yang , Y. Li , D. Wang , W. Liu , Z. Zhang , J. Liu , K. Zhang , Biomaterials 2020, 256, 120221. 32738651 10.1016/j.biomaterials.2020.120221

[106] L. Mei , G. Zhu , L. Qiu , C. Wu , H. Chen , H. Liang , S. Cansiz , Y. Lv , X. Zhang , W. Tan , Nano Res. 2015, 8, 3447. 27774139 10.1007/s12274-015-0841-8 PMC5070671

[107] L. Zhang , R. Abdullah , X. Hu , H. Bai , H. Fan , L. He , H. Liang , J. Zou , Y. Liu , Y. Sun , X. Zhang , W. Tan , J. Am. Chem. Soc. 2019, 141, 4282. 30730715 10.1021/jacs.8b10795 PMC6625512

[108] a) H. Cheng , S. Hong , Z. Wang , N. Sun , T. Wang , Y. Zhang , H. Chen , R. Pei , J. Mater. Chem. B 2018, 6, 4638; 32254408 10.1039/c8tb00758f

[109] T. Ramreddy , R. Sachidanandam , T. R. Strick , Nucleic Acids Res. 2011, 39, 4275. 21266478 10.1093/nar/gkr008 PMC3105387

[110] K. Abnous , N. M. Danesh , M. Ramezani , F. Charbgoo , A. Bahreyni , S. M. Taghdisi , Exp. Opinion Drug Del. 2018, 15, 1045. 10.1080/17425247.2018.1530656 30269603

[111] V. Bagalkot , O. C. Farokhzad , R. Langer , S. Jon , Angew. Chem. 2006, 45, 8149. 17099918 10.1002/anie.200602251

[112] W. Li , X. Yang , L. He , K. Wang , Q. Wang , J. Huang , J. Liu , B. Wu , C. Xu , ACS Appl. Mater. Interfaces 2016, 8, 25733. 27622459 10.1021/acsami.6b08210

[113] W. Szymanowski , A. Szymanowska , A. Bielawska , G. Lopez‐Berestein , C. Rodriguez‐Aguayo , P. Amero , Cancers 2023, 15, 5300. 37958473 10.3390/cancers15215300 PMC10647731

[114] M. Cao , Y. Sun , M. Xiao , L. Li , X. Liu , H. Jin , H. Pei , Chem. Res. Chin. Univ. 2020, 36, 254.

[115] P. Sun , N. Zhang , Y. Tang , Y. Yang , J. Zhou , Y. Zhao , RSC Adv. 2018, 8, 26300. 35541930 10.1039/c8ra04589e PMC9082932

[116] K. Liu , C. Xu , J. Liu , J. Mater. Chem. B 2020, 8, 6802. 32373880 10.1039/d0tb00663g

[117] B. Dai , Y. Hu , J. Duan , X. D. Yang , Oncotarget 2016, 7, 38257. 27203221 10.18632/oncotarget.9431 PMC5122387

[118] W. Ma , Y. Yang , J. Zhu , W. Jia , T. Zhang , Z. Liu , X. Chen , Y. Lin , Adv. Mater. 2022, 34, 2109609. 10.1002/adma.202109609 35064993

[119] W. Tang , L. Han , S. Duan , X. Lu , Y. Wang , X. Wu , J. Liu , B. Ding , ACS Appl. Bio Mater. 2021, 4, 7701. 10.1021/acsabm.1c00933 35006686

[120] W. Ma , Y. Yang , Z. Liu , R. Zhao , Q. Wan , X. Chen , B. Tang , Y. Zhou , Y. Lin , ACS Appl. Mater. Interfaces 2023, 15, 43359. 37670592 10.1021/acsami.3c07344

[121] C. Kielar , Y. Xin , B. Shen , M. A. Kostiainen , G. Grundmeier , V. Linko , A. Keller , Angew. Chem. 2018, 57, 9470. 29799663 10.1002/anie.201802890

[122] Z. Jiang , S. Zhang , C. Yang , J. Kjems , Y. Huang , F. Besenbacher , M. Dong , Nano Res. 2015, 8, 2170.

[123] C. E. Castro , F. Kilchherr , D. N. Kim , E. L. Shiao , T. Wauer , P. Wortmann , M. Bathe , H. Dietz , Nat. Methods 2011, 8, 221. 21358626 10.1038/nmeth.1570

[124] H. Lee , H.‐J. Kim , Tetrahedron 2014, 70, 2966.

[125] M. Singh , D. Sharma , M. Garg , A. Kumar , A. Baliyan , R. Rani , V. Kumar , Biotechnol. Adv. 2022, 61, 108052. 36307050 10.1016/j.biotechadv.2022.108052

[126] a) S. D. Perrault , W. M. Shih , ACS Nano 2014, 8, 5132; 24694301 10.1021/nn5011914 PMC4046785

[127] C. R. Lucas , P. D. Halley , A. A. Chowdury , B. K. Harrington , L. Beaver , R. Lapalombella , A. J. Johnson , E. K. Hertlein , M. A. Phelps , J. C. Byrd , C. E. Castro , Small 2022, 18, e2108063. 35633287 10.1002/smll.202108063 PMC9250639

[128] J. K. Kiviaho , V. Linko , A. Ora , T. Tiainen , E. Järvihaavisto , J. Mikkilä , H. Tenhu , Nonappa, M. A. K , Nanoscale 2016, 8, 11674. 27219684 10.1039/c5nr08355a

